# Complete Genome Analysis of Undecylprodigiosin Pigment Biosynthesizing Marine *Streptomyces* Species Displaying Potential Bioactive Applications

**DOI:** 10.3390/microorganisms9112249

**Published:** 2021-10-28

**Authors:** Chatragadda Ramesh, Maile Anwesh, Nambali Valsalan Vinithkumar, Ramalingam Kirubagaran, Laurent Dufossé

**Affiliations:** 1 National Institute of Oceanography (CSIR-NIO), Dona Paula 403004, Goa, India; 2 Atal Centre for Ocean Science and Technology for Islands, National Institute of Ocean Technology (NIOT), Ministry of Earth Sciences (MOES), Government of India (GOI), Dollygunj, Port Blair 744103, Andaman and Nicobar Islands, India; nv.vinithkumar@gmail.com; 3 Model Rural Health Research Unit (ICMR-MRHRU), Dahanu 401601, Maharashtra, India; 4 Marine Biotechnology Group, National Institute of Ocean Technology, MOES, GOI, Chennai 600100, Tamil Nadu, India; kirubagar@gmail.com; 5 Chemistry and Biotechnology of Natural Products, CHEMBIOPRO, Université de La Réunion, ESIROI Agroalimentaire, 15 Avenue René Cassin, CEDEX 9, F-97744 Saint-Denis, France

**Keywords:** prodigiosin, undecylprodigiosin, marine sediment, antioxidant, antimicrobial, type III PKS genes, bacterial genome assembly

## Abstract

Marine *Streptomyces* species are underexplored for their pigment molecules and genes. In this study, we report the genome of the undecylprodigiosin biosynthesizing gene cluster carrying *Streptomyces* sp. strain BSE6.1, displaying antioxidant, antimicrobial, and staining properties. This Gram-positive obligate aerobic bacterium was isolated from the coastal sediment of the Andaman and Nicobar Islands, India. Pink to reddish pigmented colonies with whitish powdery spores on both agar and broth media are the important morphological characteristics of this bacterium. Growth tolerance to NaCl concentrations was 2 to 7%. The assembled genome of *Streptomyces* sp. BSE6.1 contains one linear chromosome 8.02 Mb in length with 7157 protein-coding genes, 82 tRNAs, 3 rRNAs and at least 11 gene clusters related to the synthesis of various secondary metabolites, including undecylprodigiosin. This strain carries type I, type II, and type III polyketide synthases (PKS) genes. Type I PKS gene cluster is involved in the biosynthesis of red pigment undecylprodigiosin of BSE6.1, similar to the one found in the *S. coelicolor* A3(2). This red pigment was reported to have various applications in the food and pharmaceutical industries. The genome of *Streptomyces* sp. BSE6.1 was submitted to NCBI with a BioProject ID of PRJNA514840 (Sequence Read Archive ID: SRR10849367 and Genome accession ID: CP085300).

## 1. Introduction

In recent years, marine pigmented bacteria have been gaining more research interest due to the potential applications of pigment molecules in the food and drug industries [1,2,3]. Among a wide array of pigmented microbes in terrestrial and marine environments, *Streptomyces* species have gained enormous attention in biotechnological applications. Although *Streptomyces* species are well known to produce a wide range of pigments, including blue, yellow, red, orange, pink, purple, blue-green, brown, and black [1,2], prodigiosin molecules, which are red in color, are not well studied amongst the *Streptomyces* species distributed in marine milieus. 

*Streptomyces* species are known to contain a 5.1–10.1 Mbp size linear chromosome that carries core and adaptive genes [4,5]. They are spore formers with higher G+C contents (69–78%) than other Gram-positive bacteria. *Streptomyces* species are known to possess 21–45 secondary metabolites biosynthesizing gene clusters [4]. However, most of the gene clusters remain unexplored in this genus, which could have potential applications in the drug and food industry [4]. One such gene cluster is the prodigiosin biosynthetic gene cluster. Although more than 364 *Streptomyces* species are currently known [6], very few of them, such as *Streptomyces spectabilis, Streptomyces pentaticus* subsp. *jenensis* [7], *Streptoverticillium rubrireticuli, Streptomyces longispororuber* 100-19 (formerly *Streptomyces longisporus ruber*) [8], *S. spectabilis* BCC4785 [9], *Streptomyces* fusant NRCF69 [10], *Streptomyces* sp. Y-42 [11], *Streptomyces* sp. WMA-LM31 [12], *S. griseoviridis* [13], *S. lividans* [14], *Streptomyces* sp. CP1130 [15], *S. variegatus* [16], and *S. coelicolor* [17,18] are known to produce prodiginine pigments in addition to several well-studied non-actinomycetes bacteria [1].

The biosynthetic pathway of prodigiosin has been well understood in *Serratia marcescens* [19,20] and among many other prodigiosin-producing bacterial species. *S. marcescens* synthesizes prodigiosin through 33 genes, whereas *S. coelicolor* uses only 23 genes to synthesize prodigiosin derivatives [19,21]. The red gene cluster biosynthesizes prodiginines in Streptomyces species. Both *Serratia* and *Streptomyces* utilize 4-methoxy-2,2′-bipyrrole-5-carbaldehyde to synthesize prodigiosin and undecylprodigiosin, correspondingly [19,20]. Although the genome contents of several *Streptomyces* species have been reported in the last decade [4,22], the genomes of red pigment-producing *Streptomyces* species, especially marine *Streptomyces,* have remain largely uninvestigated, leaving a gap in the understanding of their evolutionary significances and drug discovery potential. Therefore, we intended to analyze and understand the genome of prodigiosin-producing *Streptomyces* BSE6.1 isolated from a coastal sediment sample.

Prodigiosin pigments are well known for their antimicrobial, anticancer, and cytotoxic properties [1,2,21,23]. Application of dried prodigiosin as a food-grade colorant in the development of prodigiosin coated microcapsules [24] and agar jellies [25] has been demonstrated from the extractions of *S. marcescens* [24], *Zooshikella* sp., and *Streptomyces* sp. [25]. Prodigiosin extracted from *Streptomyces* species has demonstrated promising antimicrobial activities against several pathogenic microbes such as *Corynebacterium bovis, Mycobacterium smegmatis, Nocardia asteroids* [7], and *Staphylococcus aureus* [7,25]. It is thought that the combined activity of antimicrobial and food colorant applications of prodigiosin would facilitate a synergistic effect in disease treatment. The present study introduces a novel species of a red-pigmented *Streptomyces* strain isolated from Andaman Islands, India’s marine environment, and its genome for industrial and biotechnological applications. The preliminary studies on prodigiosin-producing *Streptomyces* have demonstrated antimicrobial [7] and staining properties [8,25]. Although several *Streptomyces* species are known to produce a wide range of pigment compounds [1,2], the production of prodiginine derivatives by a limited number of *Streptomyces* species encouraged us to investigate the corresponding gene clusters in this *Streptomyces* sp. and compare it with other bacterial species. 

Andaman and Nicobar Islands are a chain of 836 Islands, including islands, islets and rocky outcrops, that are pristine and unexplored for microbial resources. Bio-prospecting of microbial pigments from this environment was initiated very recently [1,2,26]. The erratic weather conditions observed in this geographically distinct location appear to favor many novel pigmented microbes with potential biotechnological applications. Therefore, the present study explored the pigmented bacterial resources available in the Andaman Islands and found a potential *Streptomyces* sp. strain BSE6.1 with antibacterial and dye activity. As Andaman waters are still underexplored, we aimed to investigate the novelty of *Streptomyces* sp. strain BSE6.1 through whole-genome analysis, predict the pigment gene clusters, and compare them with those of other *Streptomyces* species genomes available in the public nucleotide databases.

## 2. Materials and Methods

A red-pigmented bacterial isolate designated as BSE6.1 was isolated from a marine sediment sample collected from Burmanallah coast (11°33′52.24″ N, 92°44′ 01.51″ E), South Andaman Islands, India. A serially diluted sediment sample was inoculated onto marine agar 2216 (Himedia, Mumbai) plates and incubated at 28 °C. After a couple of weeks, red-pigmented colonies grown were sub-cultured either on freshly prepared marine agar plates or 2% nutrient agar. Pure cultures were stored as glycerol suspensions (30%, *w*/*v*) at −20 °C for further analysis. Salt tolerance was tested on marine agar plates supplemented with various percentages of NaCl (1 to 10%), followed by streaking a pure culture, incubating at 28 °C, and measuring growth after two days. Catalase and oxidase activities were performed according to standard microbial biochemical tests [27].

**Figure 1:**
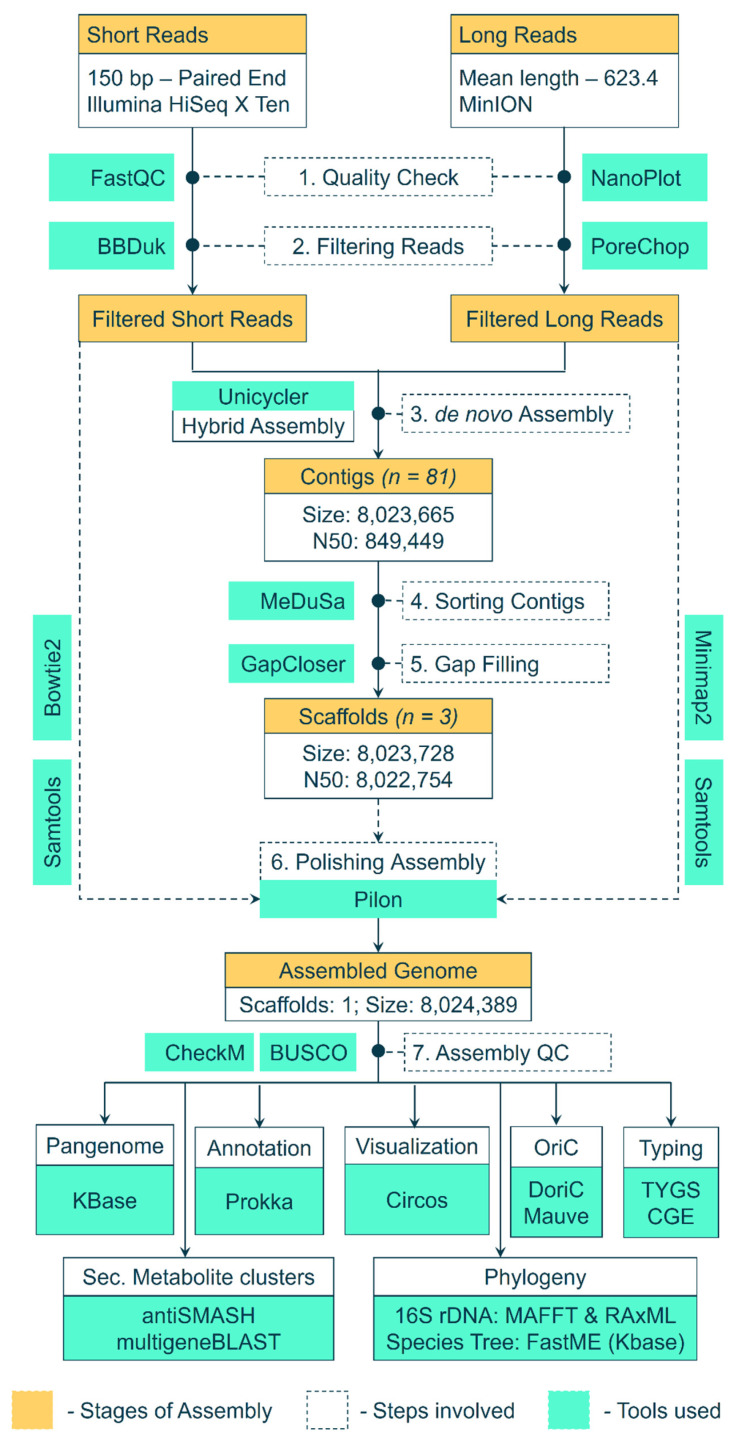
Workflow and pipeline of tools used to assemble the raw reads into a genome and further analysis of the assembled genome.

Genomic DNA of *Streptomyces* BSE6.1 was extracted using the Cetyl Trimethyl Ammonium Bromide (CTAB) and phenol–chloroform method. Extracted DNA was treated with RNase A and purified. DNA was quantified by measuring its absorbance at A260 and A280 in a NanoDrop. The Illumina Hiseq X Ten sequencing system was used to obtain 150 bp short-read paired-end raw data. In addition to these short reads, long reads were obtained using the MinIoN platform. The workflow used to assemble these raw reads and analyze the genome assembly is depicted in Figure 1. The paired-end data quality of short reads was checked using FASTQC v0.11.8 [28]. BBDuk (BBmap v38.93) was used to filter low-quality reads and adaptor sequences [29], whereas the long reads were checked with NanoPlot v1.38.1 [30] and filtered with PoreChop v0.4.8 [31]. The filtered high-quality short and long reads were assembled into contigs using a hybrid de novo assembler Unicycler v0.4.8 [32], in a de novo fashion. The 16S rRNA genes were extracted from the assembled scaffolds using Barrnap [33] and were aligned against the non-redundant nucleotide database at NCBI. The complete genome of the nearest neighbor (*Streptomyces* sp. KPB2—Accession ID: CP034353.1) [34], was used as a reference. The contigs were sorted and merged into scaffolds with the help of a reference genome using MeDusa v1.6 [35]. A gap-filling step was performed using GapCloser v1.12 [36] to generate a draft genome assembly. Furthermore, the genome assembly was polished with Pilon v1.24 [37] by mapping filtered short reads (Bowtie2 v2.4.4. [38]) and filtered long reads (minimap2 [39]) against the assembly and sorting the alignments with samtools v1.13 [40].

Genome assembly was checked for its quality using BUSCO v5.2.2 [41] and CheckM v1.1.3 [42] tools. In silico multi-locus sequence typing (MLST) of the genome was performed using the online webserver at the Centre of Genomic Epidemiology [43]. Type strain identification of the genome was performed at Type(Strain) Genome Server (TYGS) [44]. In addition to the type strain identification, a species tree was constructed with FastME [45] at KBase server [46] using 49 core Clusters of Orthologous Groups (COGs) of 200 related genomes. An additional phylogenetic tree was constructed with the 16s rRNA genes of Streptomyces species available at the Ribosomal RNA database [47]. Duplicate sequences were removed, and multiple sequence alignment (MSA) was performed using default parameters of MAFFT v7.487 for FFT-NS-I refinement method [48]. A maximum-likelihood tree was constructed based on the MSA using default parameters and 1000 bootstraps with RAxML v8.2.12 [49]. The 16s rRNA gene of Staphylococcus aureus (RefSeq ID: GCF_000013425.1) was used as an outgroup. The origin of replication (OriC) was identified using DoriC database [50] and Mauve aligner [51]. Pairwise genomic comparison of strain BSE6.1 was made with 3 other related genomes. Dotplots were constructed with minimap2 based pairwise alignment using D-Genies [52]. Prokka v1.14.6 was used to perform a local de novo annotation [53]. Pan-genome comparison with 100 related genomes (~90% 16S nucleotide identity; ~80% whole-genome aligned fraction identity) was made using the pan-genome tool at KBase server [46]. Gene clusters related to the secondary metabolite biosynthesis were identified using the antiSMASH 5.0 pipeline [54]. The red pigment-producing gene cluster of BSE6.1 was compared with that of *S. coelicolor* A3(2), *Serratia,* and *Hahella* using the multigene BLAST tool [55]. The distribution of various coding sequences (CDS) and gene clusters across the genome was plotted using Circos [56].

## 3. Results and Discussion

**Figure 2:**
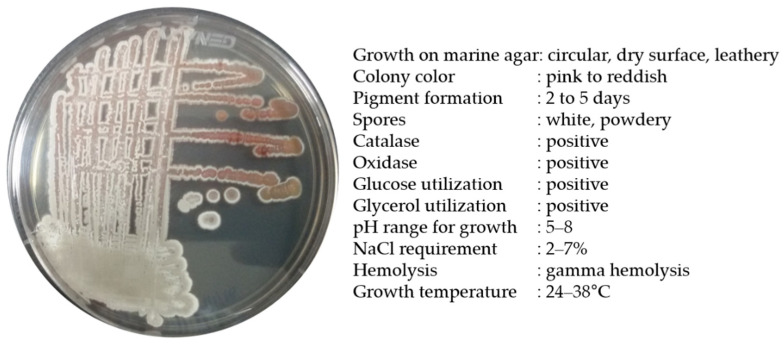
Morphological and biochemical characteristics of *Streptomyces* sp. strain BSE6.1.

Strain BSE6.1 produced a pink-colored growth in Minimal broth with 2% NaCl and red pigmentation in all other compatible media. Pale pink to reddish colonies with white powdery spores were observed after 7 or 10 days of incubation. Salt tolerance was observed up to a range of 2 to 7%. This bacterium was positive for catalase and oxidase activities. In our earlier study, strain BSE6.1 showed potential antibacterial activity against different human pathogens and also displayed a strong ability to stain epidermis and parenchyma cells of *Tridax procumbens* stem [25]. The maximum pigment production was observed at 29 °C, and the maximum temperature tolerance for its growth was 38 °C (Figure 2). The peak absorption spectrum of the red pigment of BSE6.1 was observed at 528 nm [25].

Identification of the red pigment through thin layer chromatography (TLC), Fourier-transform infrared spectroscopy (FT-IR), and proton nuclear magnetic resonance (^1^H-NMR) analyses revealed the presence of antimicrobial pigment –prodiginine derivatives in *Streptomyces* sp. BSE6.1 [25]. However, the genome analysis of strain BSE6.1 reveals the presence of an undecylprodigiosin gene cluster which is responsible for undecylprodigiosin production. Therefore, the other red fraction of *Streptomyces* strain BSE6.1 [25] is yet to be elucidated and identified through LC-MS, ^13^C NMR, HSQC, HMBC, and COSY data to confirm the production of undecylprodigiosin or related derivatives.

Previous studies reported that *Streptomyces longisporus*, *Streptomyces spectabilis* [7,57], and *Streptomyces variegatus* produce prodigiosin [16] (Table 1). However, some strains of *Streptomyces coelicolor* produce either undecylprodigiosin [17,20,58] or a mixture of prodiginine derivatives [59] (Table 1). Similar to *S. coelicolor* [17,20,58,59], the first fraction of red pigment eluted from *Streptomyces* strain BSE6.1 through TLC revealed the presence of methyl-3-propyl prodiginine and 2-methyl-3-butyl prodiginine in mass spectrometry analysis but identified it as prodigiosin in ^1^H NMR analysis [25]. Methyl-3-propyl prodiginine and 2-methyl-3-butyl prodiginine were also identified in actinomycetes [60], non-actinomycetes bacteria such as *Pseudoalteromonas rubra* [61], and *Serratia marcescens* [62]. These studies suggest that some strains of *Streptomyces* produce either prodigiosin or undecylprodigiosin, whereas some produce a mixture of prodiginine analogs.

**Figure 3:**
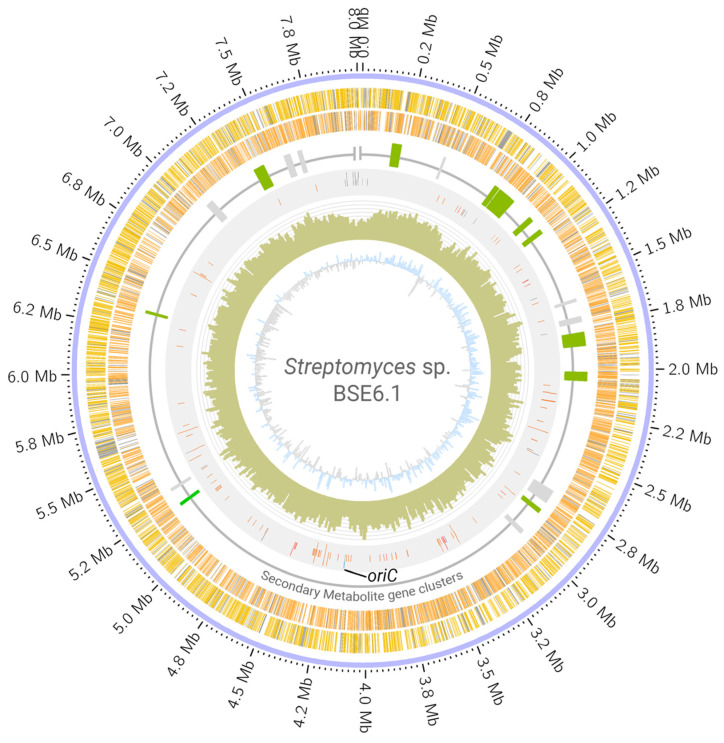
Circular visualization of *Streptomyces* sp. strain BSE6.1 genome. The scaffold is represented in the outer circle. The scaffold is followed by coding regions (CDS) in the sense (yellow bands) and anti-sense (orange bands) directions. Grey bands represent hypothetical CDS. The third circle represents the distribution of gene clusters coding for secondary metabolites (green: clusters which are >75% similar to those present in related organisms; grey: <75% similarity). The fourth circle represents the RNA genes (orange), transposases (grey), phage genes (purple) dnaA gene (blue), and oriC region (green and labelled). Histograms in the fifth circle indicate the GC content per 10,000 bases. The innermost circle represents GC skew data per 10,000 bases (blue indicates positive skewness and grey negative skewness).

Whole-genome sequencing of strain BSE6.1 produced a total of 7,528,288 reads. Assembling these raw reads resulted in a single scaffold of 8.02 Mb with no extra-chromosomal content. Annotating the assembled genome of strain BSE6.1 indicated the presence of at least 7157 protein-coding genes, 82 tRNA coding genes, 3 rRNA coding genes, and 1 responsible for the production of tmRNA (Table 2, Figure 3). Subsystem coverage of the identified CDS was 19%, involving nearly 324 subsystem types (Figure S1). Subsystems with the highest coverage of genes/features include amino acid, carbohydrate, protein, and vitamin metabolic pathways. Furthermore, at least 43 genes were involved in defense mechanisms such as resistance to antibiotics and toxic compounds. In addition, at least 11 gene clusters involved in the synthesis of other secondary metabolites were also identified (Figure S2). Most members of the Streptomyces genus have linear chromosomes [4,5] and strain BSE6.1 is not an exception. There are no overlapping 5′ and 3′ ends in the scaffold, indicating its non-circular configuration. Furthermore, the *oriC* region and *dnaA* gene were identified approximately at the center of the scaffold, similar to that of *S. coelicolor* A3(2) (Figure 3).

**Figure 4:**
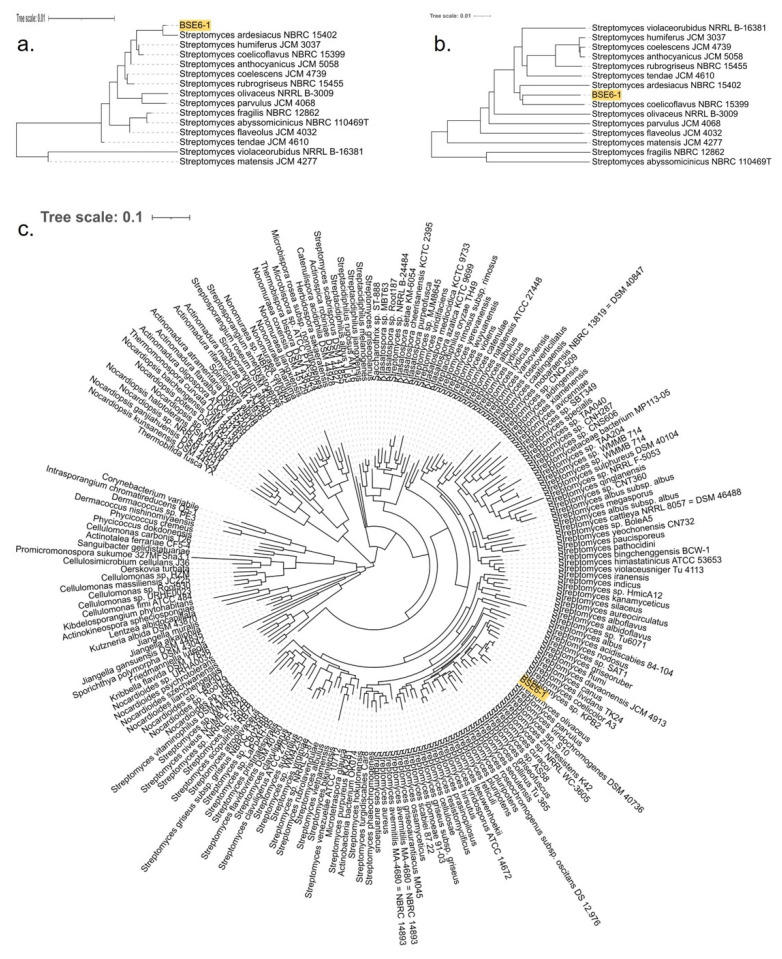
GBDP tree with 100 bootstraps for (**a**) 16S rRNA genes and (**b**) genomes of strain BSE6.1 along with 14 type strains with highest dDDH (d4) similarity. (**c**) Species tree constructed using 49 core/conservative COGs of strain BSE6.1 and 200 related/homologous genomes with at least 90% 16S nucleotide identity and ~80% whole-genome aligned fraction identity.

BLAST analysis based on the 16s rRNA sequences suggested that strain BSE6.1 had a 99.71% similarity with various unclassified *Streptomyces* species available in the GenBank. The most similar strains include *Streptomyces* sp. NA03103 (isolated from marine sediment in China) (GenBank: CP054920), *Streptomyces* sp. strain HB-N217 (isolated from a marine sponge, *Forcepia* sp. in the USA) [77], *Streptomyces* sp. CCM_MD2014 (soil isolate from the USA) [78], *Streptomyces* sp. KPB2 (isolated from the pollen of kiwi fruit from South Korea) [34], *Streptomyces* sp. PM-R01 (isolated from Durian fruit, *Durio zibethinus*, in Thailand) (GenBank: LC381944), and *Streptomyces* sp. IT-M01 (isolated from a sea crab, *Thalamita crenata*, in Thailand) (GenBank: LC386952). Furthermore, 16S rRNA genes of BSE6.1 and 208 *Streptomyces* species were used to construct a phylogenetic tree (Figure S3). The strain typing of BSE6.1 at TYGS indicated no available type strain, which is closely related to the query genome. The highest pairwise digital DNA–DNA hybridization similarity (dDDH, d4 value corresponding to the sum of all identities found in HSPs divided by overall HSP length) was 48.7% with type strain *Streptomyces coelicoflavus* NBRC 15399 (Sup. Data 1). A genome blast distance phylogenetic (GBDP) tree was constructed for BSE6.1 and the related type strains using 16S rRNA gene and complete genome data (Figure 4a,b). In addition to detecting the closest type strain, a species tree was constructed using 49 core COGs in related genomes [46] (Sup. Data 2). In the species tree, BSE6.1 clustered with the strains *viz. Streptomyces* sp. KPB2, *S. coelicolor* A3(2), *S. lividans* TK24, *S. olivaceus*, *S. parvulus*, etc (Figure 4c).

**Figure 5:**
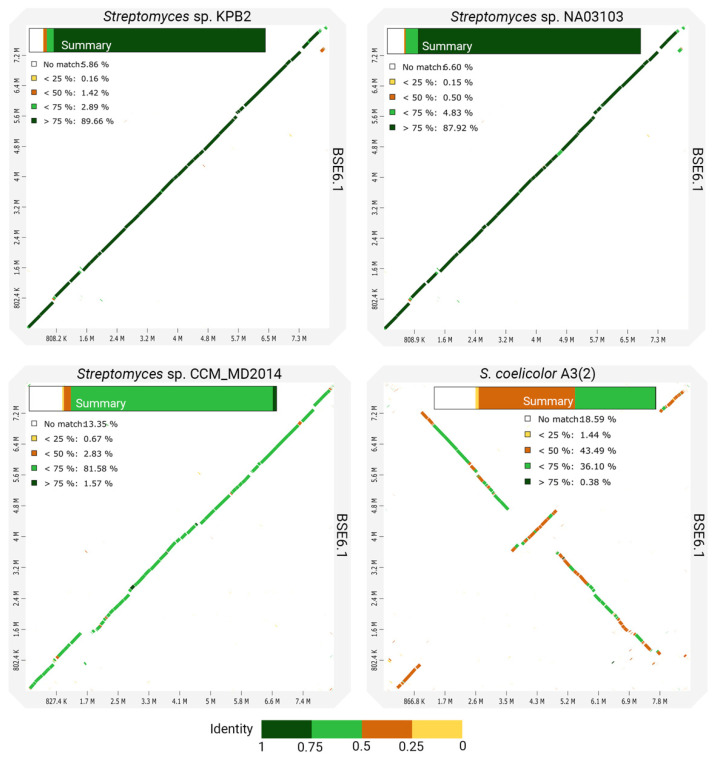
Dotplots showing the pairwise comparisons of strain BSE6.1 genome and three closely related genomes. A summary of each comparison is shown within the corresponding plot.

However, the whole-genome comparison of BSE6.1 with other closely related species shows many variations in its genomic content (Figure 5). In concordance with the phylogenetic distances, the genomes of strain KPB2 and strain NA03103 have the most similar genomic regions with BSE6.1. Comparatively less identical homologous regions were observed while comparing BSE6.1 with strain CCM_MD2014. Another comparison of BSE6.1 with one of the well-studied pigment-producing bacteria, *S. coelicolor* A3(2) [70], presented the least identical synteny among the four comparisons. Furthermore, the in silico MLST analysis of the BSE6.1 genome revealed the presence of a novel allelic profile—16S_99, atpD_185, gyrB_124, recA_156, rpoB_175 and trpB_190 (Table 3). All the in silico analyses suggested that the strain BSE6.1 could be a novel species of *Streptomyces*. However, further phenotypic characterizations are needed to confirm its novelty.

**Figure 6:**
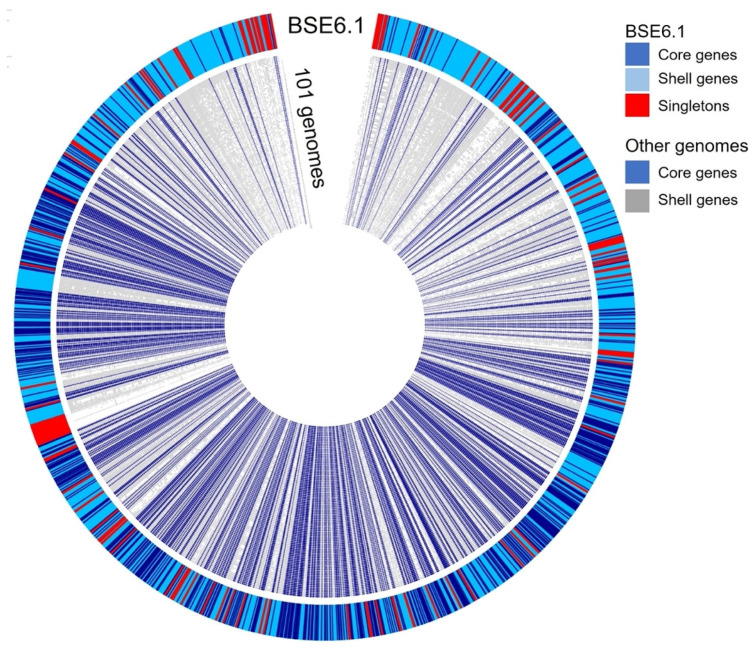
Pangenome comparison of strain BSE6.1 and 101 related genomes (Sup. Data 4) of Streptomycetaceae family. The genome of strain BSE6.1 has 12.6% of conserved genes, 84.1% of shared or shell genes, and 3.3% of unique genes.

A pan-genomic comparison was made between 101 related genomes belonging to the Streptomycetaceae family and that of strain BSE6.1 (Figure 6). A total of 720,604 translated genes belong to 123,491 homologous gene families were identified. Out of these, 726 families were conserved across the genomes, 41,274 were shell gene families, and 81,497 were singletons. Strain BSE6.1 has 7157 genes, of which 902 belong to the core gene cluster, 6016 genes belonging to the shell gene cluster, and 239 genes are unique to BSE6.1. The genes confined to strain BSE6.1 are mostly hypothetical (184 out of 239 genes), apart from some interesting genes viz. serine protease genes (perform physiological roles), MarR family (responsible for multiple antibiotic resistance), SsgA sporulation regulator, etc (Sup. Data 3).

*Streptomyces* species are ubiquitous in nature, with more than 500 *Streptomyces* species reported from various environments such as terrestrial, coastal, deep-sea, deserts, and polar regions [6]. Under unfavorable conditions, these species produce external hyphae, which divide into spores. *Streptomyces* species possess antibiotic resistance genes; thus, they display potential bioactive properties. Many species of *Streptomyces* are known to produce secondary metabolites, antibiotics [79,80], and very few *Streptomyces* species are known to produce pigments such as prodigiosin derivatives having antimicrobial and anticancer properties [1,6,19]. The genome analysis of BSE6.1 revealed the presence of 23 gene clusters responsible for the production of ectoine, polyketides, etc (Figure S2). Out of these 23 clusters, at least 11 showed >75% similarity with existing gene clusters of different strains (Figures S4 and S5). The information about all the other gene clusters and their similarity to the other Streptomyces may be accessed through anti-smash (Sup. Data 5).

The genome of BSE6.1 contains three types of PKSs, namely type I, type II, and type III. Strain BSE6.1 has two copies of type III polyketide synthase (PKS) genes observed in clusters 20 and 21, coding for herboxidiene, an antitumor molecule reported in *Streptomyces* sp. [81], and germicidin, which is responsible for the development of spore formation and aerial hyphae elongation [82], respectively. The type III PKS genes in *Streptomyces* species are known to produce red to brownish pigments with potential antimicrobial and antioxidant activities [83,84]. Cluster 13 represents a type II PKS, which is responsible for grey-pink spore pigmentation in *Streptomyces* species [85,86].

**Figure 7:**
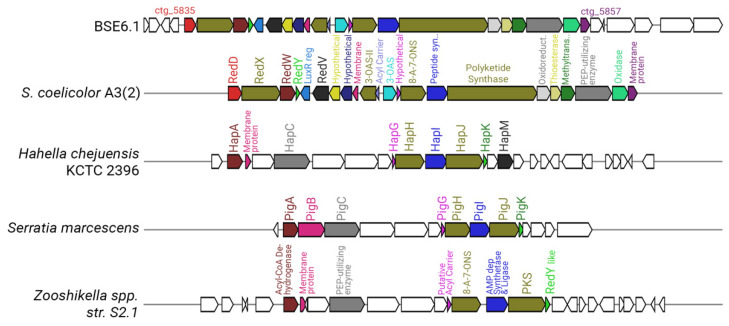
Comparative representation of the undecylprodigiosin cluster in BSE6.1 (cluster 10 of Supplementary Figure S2) with that of *S. coelicolor* A3(2), *Hahella chejuensis* KCTC 2396, *Serratia marcescens,* and *Zooshikella* sp. S2.1. (Genes represented without any color in the strain BSE6.1 have unknown functions, whereas those in the other species have no homologs in BSE6.1).

Strain BSE6.1 has a type I PKS system in cluster 10, which is responsible for undecylprodigiosin production. The prodigiosin biosynthesis gene cluster was identified as *pig* gene cluster in *Serratia marcescens* [19,87]. Prodigiosin synthesizing genes in *Hahella chejuensis* KCTC 2396 and *Pseudoalteromonas* species were identified as *hap* gene cluster [88], while *red* gene cluster was identified for undecylprodigiosin biosynthesis in *S. coelicolor* A3(2) [58]. The prodigiosin biosynthesizing cluster found in the *Streptomyces* species is the largest cluster, with 23 genes (Figure 7). Cluster 10 of strain BSE6.1 showed 100% similarity with *red* gene cluster of *S. coelicolor* A3(2). Cluster 10 has 32 genes, of which 23 genes are responsible for the production of the bioactive red pigment undecylprodigiosin. This cluster comprises regulatory genes, core, and additional genes involved in the biosynthesis of prodigiosin derivatives, similar to that of *red* gene cluster of *S. coelicolor* A3(2).

Cluster 19 displayed 100% similarity with ectoine biosynthesizing genes, indicating that BSE6.1 is capable of producing ectoine—an anticancer molecule [89] that regulates osmotic stress [90] and acts as a stress protectant against various environmental stresses [91]. Clusters 7 and 17 displayed siderophore genes that were involved in the production of enduracidin and desferrioxamine B, respectively. Desferrioxamine B is a drug used to treat iron overload disease in humans [92,93], while enduracidin is known to display antibiotic activity [94]. Cluster 14 represents albaflavenone, an antibiotic terpene molecule produced by *S. coelicolor* A3(2) [95,96]. Chemical molecules produced by *S. coelicolor* A3(2) such as germicidin [82], ectoine [91], albaflavenone [95], coelichelin [97], hopanoids [98], sapB protein [99], and coelibactin [100] are observed in *Streptomyces* strain BSE6.1 with a 100% similarity match. Ashimides molecules produced by *Streptomyces* sp. NA03103 [101] are not detected in *S. coelicolor* A3(2), but *Streptomyces* strain BSE6.1 shows 100% similarity with ashimides synthesizing gene.

Interestingly, the genome content of strain BSE6.1 is distinct from other *Streptomyces* species. It is an important evolutionary aspect that these related and non-related bacterial lineages are capable of producing a variety of prodiginine analogs for their defensive function in the surrounding milieus. As studies on the diversity and distribution of marine pigmented *Streptomyces* species are scarce, further research on this aspect would provide new insights into the evolutionary spread and species distribution of pigmented *Streptomyces* in different environments. We infer that pigment gene clusters of microbes such as *Streptomyces* may serve as an evolutionary marker to address the actual place of origin and spread of prodiginine pigments in the marine or terrestrial milieus during the evolutionary process. The variability in the whole genome content and novel alleles in the MLST profile indicate its status as a novel species. Thus, based on complete genome analysis, we propose strain BSE6.1 as *Streptomyces prasanthi* sp. nov. This study provides the whole genome of *Streptomyces* sp. BSE6.1 for further comparative studies with other *Streptomyces* species on taxonomical, evolutionary, and biotechnological aspects. As it is the first ever mined genome of prodigiosin-producing marine *Streptomyces* BSE6.1, it would serve as a reference genome for comparative studies to predict the novelty of the genomic contents of other *Streptomyces* species and non-*Streptomyces* species. 

## Figures and Tables

**Table 1:** Details of various *Streptomyces* species capable of producing prodiginine derivatives.

Species	Origin	Pigment	NMR Spectra	References
*Streptomyces* sp. BSE6.1	Marine sediment	Prodigiosin-like	3.430 ppm, 3.252 ppm	[25], this study
*Streptomyces* fusant NRCF69	Marine sponge *Latrunculia corticata*	Prodigiosin-like		[10]
*Streptomyces* sp. UKMCC_PT15	Seawater	Undecylprodigiosin	6.21, 6.84, 7.00, 6.09 6.95 ppm	[63]
*Streptomyces* sp. SCSIO 11594	Deep-sea sediment	Undecylprodigiosin and metacycloprodigiosin		[64]
*Streptomyces* sp. CP1130	Soil	Undecylprodigiosin, Metacycloprodigiosin		[15]
*Streptomyces spectabilis* L20190601	Lake soil	Metacycloprodigiosin		[65]
*Streptomyces* sp. JS520	Cave soil	Undecylprodigiosin		[66]
*Streptomyces* strain MBK6	Soil	Undecylprodigiosin, Metacycloprodigiosin		[67]
*Streptomyces* sp. strains NP2 and NP4	Soil	Prodigiosin-like		[68]
*Streptomyces* sp. WMA-LM31	Desert soil	Prodigiosin		[12]
*Streptomyces* sp. Y-42	Leaf and grass compost	Undecylprodiginine and butylcycloheptylprodiginine		[11]
*Streptomyces* sp. JAR6	Leaf litter soil	Undecylprodigiosin	7.50, 6.68, 6.35, 6.05, 5.39, 5.37, 5.30, 5.12, 3.80, 2.48, 1.31, 0.88 ppm	[69]
*Streptomyces coelicolor* A3(2)	Soil	Undecylprodiginine		[70]
*S. coelicolor* A3(2)	Soil	Undecylprodigiosin		[17,18]
*S. coelicolor* A3(2)	Soil	Undecylprodiginine and butylcycloheptylprodiginine		[58]
*S. coelicolor* A3(2)	Soil	Undecylprodigiosin	1.24, 1.33, 2.85, 4.01, 6.20, 6.34, 6.55, 6.76, 6.90, 6.97, 7.23 ppm	[59]
*S. coelicolor* A3(2)	Soil	Butyl-meta-cycloheptylprodiginine	0.9, 1.2, 2.3, 3.1, 3.9 ppm	[59]
*S. coelicolor* M145	Soil	Undecylprodigiosin		[71]
*Streptomyces griseoviridis* 2464-S5		Prodigiosin R1 & R2		[13,72,73]
*Streptomyces lividans*		Undecylprodigiosin		[14]
*S. lividans*		Undecylprodigiosin		[74]
*Streptomyces longispororuber*		Metacycloprodigiosin		[7,8]
*S. longispororuber* IMRU 3762		Undecylprodiginine and metacycloprodigiosin		[11]
*S. longispororuber*		Metacycloprodigiosin		[75]
*S. longispororuber* DSM 40599		Undecylprodiginine and metacycloprodigiosin		
*Streptomyces parvulus*		Butylcycloheptylprodigiosin and undecylprodiginine	5.5–7.5, 4.0, 2.2, 1.1–1.5, and 0.86 ppm	[76]
*Streptomyces pentaticum* subsp. *jenensis*		Prodiginine		[7]
*Streptomyces spectabilis*		Prodiginine		[7]
*Streptomycesspectabilis* BCC 4785		Metacycloprodigiosin		[9]
*Streptomyces variegatus*		Prodigiosin		[16]
*Streptoverticillium rubrireticuli* 100-19	Soil	Undecylprodiginine and butylcycloheptylprodiginine		[8]

**Table 2:** Features of *Streptomyces* sp. strain BSE6.1 genome.

Genome Features	Chromosome 1
Genome size (bp)	8,024,389
G+C content (%)	72.25
Contigs	1
Longest Contig	8,024,389
Total number of CDS	7157
Total hypothetical genes	1193
tRNA	82
rRNA	3
tmRNA	1
Number of Functional Subsystems	324
Number of gene clusters responsible for secondary metabolite production	23 (11 have more than 75% similarity with known clusters)
BUSCO: C:99.8% [S:99.5%,D:0.3%], F:0.1%, M:0.1%, n:1579	
Total BUSCO groups searched (n)	1579
Complete BUSCOs (C)	1575
Complete & single copy BUSCOs (S)	1571
Complete & duplicated BUSCOs (D)	4
Fragmented BUSCOs (F)	1
Missing BUSCOs (M)	3
CheckM	
Completeness	100%
Contamination	0.14%
Strain heterogeneity	0%

**Table 3:** MLST profile of *Streptomyces* sp. strain BSE6.1 genome.

Locus	Identity	Coverage	Alignment Length	Allele Length	Allele
16S	98.87	99.7	1338	1336	16S_99
*atpD*	99.59	100	495	495	atpD_185
*gyrB*	98.27	100	405	405	gyrB_124
*recA*	98.01	100	504	504	recA_156
*rpoB*	98.51	100	540	540	rpoB_175
*trpB*	97.17	100	567	567	trpB_190

## Data Availability

Genome sequence of *Streptomyces* BSE6.1 is submitted in Sequence Read Archive (SRA) under Bioproject: PRJNA514840. The BioSample accession ID of strain BSE6.1 is SAMN12598824. Genome assembly was submitted and is under process (SUB10526264).

## Supplementary Materials

The following are available online at https://www.mdpi.com/article/10.3390/microorganisms9112249/s1, Figure S1: Subsystems, Figure S2: Clusters of BSE6.1, Figure S3: 16S rRNA based phylogenetic tree, Figures S4 and S5: Clusters in detail, Sup. Data 1: TYGS summary, Sup. Data 2: Core COGs used in the construction of species tree, Sup. Data 3: Unique genes of BSE6.1, Sup. Data 4: List of genomes, Sup. Data 5: All clusters and their similarity to the other *Streptomyces*.
