# Supplementary material for: Complete Genome Analysis of Undecylprodigiosin Pigment Biosynthesizing Marine Streptomyces Species Displaying Potential Bioactive Applications
Source: Microorganisms. 2021 Oct 28;9(11):2249. doi: 10.3390/microorganisms9112249 (PMC8618203; doi:10.3390/microorganisms9112249)
Supplement: Supplementary file 1 [file microorganisms-09-02249-s001.zip › Sup Data 1.pdf]

TYPE (STRAIN) GENOME SERVER

JOB SUMMARY

PRINT DATE:	2021-10-11 13:48:18 +0200
JOB ID:	fd600293-3756-4483-aa2c-4c100c742212
RESULT PAGE:	https://tygs.dsmz.de/user_results/show?guid=fd600293-3756-4483-aa2c-4c100c742212

Table 1: Phylogenies

Publication-ready versions of both the genome-scale GBDP tree and the 16S rRNA gene sequence tree can be customized and exported either in SVG (vector graphic) or PNG format from within the phylogeny viewers in your TYGS result page. For publications the **SVG format is recommended** because it is lossless, always keeps its high resolution and can also be easily converted to other popular formats such as PDF or EPS. Please follow the link provided above!

Table 2: Identification

The below list contains the result of the TYGS species identification routine.

Explanation of remarks that might occur in the below table:

remark [R1]: The TYGS type strain database is automatically updated on an almost daily basis. However, if a particular type strain genome is not available in the TYGS database, this can have several reasons which are detailed in the FAQ. You can request an extended 16S rRNA gene analysis via the 16S tree viewer found in your result page to detect **not yet genome-sequenced** type strains relevant for your study.

remark [R2]: > 70% dDDH value (formula d_4) and (almost) minimal dDDH values for gene-content formulae d_0 and d_6 indicate a potentially unreliable identification result and should thus be checked via the 16S rRNA gene sequence similarity. Such strong deviations can, in principle, be caused by sequence contamination.

remark [R3]: G+C content difference of > 1 % indicates a potentially unreliable identification result because within species G+C content varies no more than 1 %, if computed from genome sequences (PMID: 24505073).

Strain	Conclusion	Identification result	Remark
**'BSE6-1'**	potential new species		see [R1]

Table 3: Pairwise comparisons of user genomes vs. type-strain genomes

The following table contains the pairwise dDDH values between your user genomes and the selected type-strain genomes. The dDDH values are provided along with their confidence intervals (C.I.) for the three different GBDP formulas:

• formula d_0 (a.k.a. GGDC formula 1): length of all HSPs divided by total genome length
• formula d_4 (a.k.a. GGDC formula 2): sum of all identities found in HSPs divided by overall HSP length
• formula d_6 (a.k.a. GGDC formula 3): sum of all identities found in HSPs divided by total genome length

Note: Formula d_4 is independent of genome length and is thus robust against the use of incomplete draft genomes. For other reasons for preferring formula d_4, see the FAQ.

Query	Subject	d_0	C.I. d_0	d_4	C.I. d_4	d_6	C.I. d_6	Diff. G+C Percent
**'BSE6-1.fasta'**	*Streptomyces coelicoflavus* NBRC 15399	64.4	[60.7 - 68.1]	48.7	[46.1 - 51.4]	62.3	[59.0 - 65.5]	0.07
**'BSE6-1.fasta'**	*Streptomyces ardesiacus* NBRC 15402	62.0	[58.3 - 65.6]	46.6	[44.0 - 49.2]	59.6	[56.4 - 62.8]	0.41
**'BSE6-1.fasta'**	*Streptomyces tendae* JCM 4610	58.7	[55.1 - 62.3]	45.9	[43.3 - 48.4]	56.7	[53.5 - 59.8]	0.3
**'BSE6-1.fasta'**	*Streptomyces rubrogriseus* NBRC 15455	64.4	[60.6 - 68.0]	45.8	[43.2 - 48.4]	61.3	[58.0 - 64.5]	0.01
**'BSE6-1.fasta'**	*Streptomyces coelescens* JCM 4739	63.5	[59.7 - 67.1]	45.5	[42.9 - 48.0]	60.5	[57.2 - 63.7]	0.0
**'BSE6-1.fasta'**	*Streptomyces anthocyanicus* JCM 5058	66.7	[62.9 - 70.4]	45.5	[43.0 - 48.1]	63.2	[59.8 - 66.4]	0.09
**'BSE6-1.fasta'**	*Streptomyces humiferus* JCM 3037	58.9	[55.3 - 62.5]	45.3	[42.7 - 47.8]	56.6	[53.5 - 59.8]	0.32
**'BSE6-1.fasta'**	*Streptomyces violaceorubidus* NRRL B-16381	56.1	[52.5 - 59.6]	44.4	[41.9 - 47.0]	54.0	[50.9 - 57.1]	0.02
**'BSE6-1.fasta'**	*Streptomyces olivaceus* NRRL B-3009	45.1	[41.7 - 48.5]	36.4	[34.0 - 39.0]	42.7	[39.7 - 45.7]	0.16
**'BSE6-1.fasta'**	*Streptomyces parvulus* JCM 4068	50.7	[47.3 - 54.1]	34.9	[32.5 - 37.4]	46.6	[43.6 - 49.7]	0.56
**'BSE6-1.fasta'**	*Streptomyces flaveolus* JCM 4032	47.7	[44.3 - 51.1]	33.5	[31.1 - 36.0]	43.8	[40.8 - 46.8]	0.01
**'BSE6-1.fasta'**	*Streptomyces matensis* JCM 4277	36.2	[32.8 - 39.7]	26.2	[23.9 - 28.7]	32.7	[29.8 - 35.8]	0.37
**'BSE6-1.fasta'**	*Streptomyces abyssomicinicus* NBRC 110469T	21.0	[17.8 - 24.7]	24.2	[21.8 - 26.6]	20.5	[17.8 - 23.6]	0.88
**'BSE6-1.fasta'**	*Streptomyces fragilis* NBRC 12862	21.7	[18.5 - 25.4]	24.0	[21.7 - 26.5]	21.1	[18.3 - 24.1]	0.96

Table 4: Strains in your dataset

Joint dataset of automatically determined closest type strains (if this mode was chosen), manually selected type strains (if selected accordingly) and the provided user strains, if provided (marked in yellow).

Strain	Authority	Other deposits	Synonyms	Base pairs	Percent G+C	No. proteins	Goldstamp	Bioproject accession	Biosample accession	Assembly accession	IMG OID
Streptomyces abyssomicinicus NBRC 110469T	Komaki et al. 2020	CHI39	*Streptomyces abyssomicinicus*	7911 144	73.1	6697		PRJDB4097	SAMD00035864	GCA_008974245	
Streptomyces fragilis NBRC 12862	Anderson et al. 1956	BCRC 13654; CCRC 13654; NRRL 2424; NRRL ISP-5044; ATCC 23908; DSM 40044; JCM 4187; JCM 4638; IFO 12862; VKM Ac-1773; CBS 804.68; HAMBI 1083; HAMBI 1090; IMET 43575; NCIB 9795; NCIMB 9795; P-D 04926; RIA 1111	*Streptomyces fragilis*	6843 555	73.2	5883	Gp0367879	PRJDB6181	SAMD00090286	GCA_003112515	
Streptomyces rubrogriseus NBRC 15455	(ex Krassilnikov 1970) Terekhova 1986	ATCC 43691; DSM 41477; JCM 6927; IFO 15455; IFO (now NBRC) 15455; VKM Ac-1216; INA 2626	*Streptomyces rubrogriseus*	8448 184	72.2	7574		PRJDB6185	SAMD00090290	GCA_003112595	

Strain	Authority	Other deposits	Synonyms	Base pairs	Percent G+C	No. proteins	Goldstamp	Bioproject accession	Biosample accession	Assembly accession	IMG OID
Streptomyces coelicoflavus NBRC 15399	(ex Ryabova and Preobrazhenskaya 1957) Terekhova 1986	DSM 41471; IFO 15399; IFO (now NBRC) 15399; INA 9630	*Streptomyces coelicoflavus*	8722322	72.2	7739	Gp0367877	PRJDB6183	SAMD00090288	GCA_003112555	
Streptomyces ardesiacus NBRC 15402	(Baldacci et al. 1955) Komaki and Tamura 2020	NRRL B-1773; DSM 40934; JCM 5815; IFO 15402; AS 4.1682; CGMCC 4.1682; CBS 100.56	*Actinomyces diastaticus* subsp. *ardesiacus*; *Streptomyces ardesiacus*; *Streptomyces diastaticus* subsp. *ardesiacus*	7843316	72.7	6894	Gp0367878	PRJDB6184	SAMD00090289	GCA_003112575	
Streptomyces matensis JCM 4277	Margalith et al. 1959	NRRL B-2576; NRRL ISP-5188; ATCC 23935; DSM 40188; JCM 4268; JCM 4651; IFO 12889; NBRC 12889; CBS 919.68; HAMBI 1048; IMET 42065; NCIB 9826; NCIMB 9826; RIA 1142; RIA 570	*Streptomyces matensis*	7325782	72.6	6731		PRJDB10510	SAMD00245408	GCA_014649175	

Strain	Authority	Other deposits	Synonyms	Base pairs	Percent G+C	No. proteins	Goldstamp	Bioproject accession	Biosample accession	Assembly accession	IMG OID
Streptomyces tendae JCM 4610	Ettlinger et al. 1958	LMG 19314; LMG 5987; BCRC 12167; CCRC 12167; IFM 1176; NRRL B-2313; NRRL ISP-5101; ATCC 19812; DSM 40101; JCM 4149; IFO 12822; NBRC 12822; AS 4.1460; CGMCC 4.1460; VKM Ac-1889; CBS 565.68; IMET 40459; NCIB 9614; NCIMB 9614; RIA 1092; RIA 534; UNIQEM 199	*Streptomyces tendae*	8799342	72.0	8163		PRJDB10510	SAMD00245486	GCA_014650435	

Strain	Authority	Other deposits	Synonyms	Base pairs	Percent G+C	No. proteins	Goldstamp	Bioproject accession	Biosample accession	Assembly accession	IMG OID
Streptomyces flaveolus JCM 4032	(Waksman 1923) Waksman and Henrici 1948	LMG 19328; BCRC 12489; CCRC 12489; KCTC 9022; NRRL B-1334; NRRL B-2688; NRRL ISP-5061; ATCC 19754; ATCC 3319; CECT 3181; DSM 40061; DSM 40328; JCM 4577; IFO 3408; IFO 3715; IFO 12768; NBRC 3408; NBRC 3715; NBRC 12768; CCM 3171; AS 4.1432; CGMCC 4.1432; VKM Ac-965; CBS 128.20; CBS 493.68; HAMBI 893; IMET 40233; RIA 1035; RIA 485; UNIQEM 141	*Actinomyces flaveolus*; *Streptomyces flaveolus*	7862 229	72.2	7259		PRJDB10510	SAMD00245381	GCA_014648815	

Strain	Authority	Other deposits	Synonyms	Base pairs	Percent G+C	No. proteins	Goldstamp	Bioproject accession	Biosample accession	Assembly accession	IMG OID
Streptomyces anthocyanicus JCM 5058	(Krassilnikov et al. 1965) Pridham 1970 emend. Komaki 2021	KCTC 9755; NRRL B-12341; ATCC 19821; DSM 41422; IFO 14892; IFO (now NBRC) 14892; NBRC 14892; AS 4.1683; CGMCC 4.1683	*Actinomyces anthocyanicus*; *Streptomyces anthocyanicus*	8163 317	72.3	7425		PRJDB10510	SAMD00245547	GCA_014651155	
Streptomyces coelescens JCM 4739	(Krassilnikov et al. 1965) Pridham 1970	NRRL B-12348; NRRL-ISP 5421; ATCC 19830; DSM 40421; IFO 13378; IFO (now NBRC) 13378; NBRC 13378; AS 4.1594; CGMCC 4.1594; VKM Ac-98; CBS 679.72; INMI 20-41; NCIB 10042; NCIMB 10042; RIA 1339	*Actinomyces coelescens*; *Streptomyces coelescens*	8636 598	72.2	7923		PRJDB10510	SAMD00245508	GCA_014650795	

Strain	Authority	Other deposits	Synonyms	Base pairs	Percent G+C	No. proteins	Goldstamp	Bioproject accession	Biosample accession	Assembly accession	IMG OID
Streptomyces humiferus JCM 3037	Goodfellow et al. 1986	IFM 1139; KCTC 9116; ATCC 15719; ATCC 15812; DSM 43030; IFO 12244; IFO (now NBRC) 12244; NBRC 12244; AS 4.1070; CGMCC 4.1070; VKM Ac-644; IMET 43409; KCC A-0037; NCIMB 10164; RIA 729	*Actinopycnidium caeruleum*; *Streptomyces humiferus*	9225795	71.9	8527		PRJDB10510	SAMD00245295	GCA_014647695	

Strain	Authority	Other deposits	Synonyms	Base pairs	Percent G+C	No. proteins	Goldstamp	Bioproject accession	Biosample accession	Assembly accession	IMG OID
Streptomyces parvulus JCM 4068	Waksman and Gregory 1954	LMG 19312; BCRC 12046; CCRC 12046; NRRL B-1628; NRRL-ISP 5048; ICMP 156; ATCC 12434; ATCC 19796; DSM 40048; JCM 4601; IFO 13193; NBRC 13193; VKM Ac-1063; CBS 418.59; CBS 548.68; HUT 6081; IMET 41380; NCIB 11240; NCIMB 11240; RIA 1075; RIA 507; UNIQEM 182	*Streptomyces parvulus*	7663 338	72.8	6933		PRJDB10510	SAMD00245384	GCA_014648855	
Streptomyces violaceorubidus NRRL B-16381	Terekhova 1986 emend. Nouioui et al. 2018	LMG 20319; ATCC 43697; DSM 41478; JCM 6931; IFO 15463; NBRC 15463; VKM Ac-1292; INA 770	*Streptomyces violaceorubidus*	7651 467	72.2	6730	Gp0188077	PRJNA224116	SAMN02645210	GCF_000717995	

Strain	Authority	Other deposits	Synonyms	Base pairs	Percent G+C	No. proteins	Goldstamp	Bioproject accession	Biosample accession	Assembly accession	IMG OID
Streptomyces olivaceus NRRL B-3009	(Waksman 1923) Waksman and Henrici 1948 emend. Nouioui et al. 2018	BCRC 11485; CCRC 11485; NRRL B-1224; NRRL ISP-5072; ATCC 19794; ATCC 3335; CCUG 11111; DSM 40072; JCM 4402; IFO 12805; NBRC 12805; CCM 3188; VKM Ac-254; CBS 546.68; INA 3200; RIA 1073; RIA 481; UNIQEM 180	*Actinomyces olivaceus*; *Streptomyces olivaceus*	8584 392	72.4	7445	Gp0187965	PRJNA224116	SAMN02645259	GCF_000721235	
BSE6-1.fasta				8024 389	72.3	7173					

Methods, Results and References

The genome sequence data were uploaded to the Type (Strain) Genome Server (TYGS), a free bioinformatics platform available under https://tygs.dsmz.de, for a whole genome-based taxonomic analysis [1]. The results were provided by the TYGS on 2021-10-10. The TYGS analysis was subdivided into the following steps:

Determination of closely related type strains

Determination of closest type strain genomes was done in two complementary ways: First, all user genomes were compared against all type strain genomes available in the TYGS database via the MASH algorithm, a fast approximation of intergenomic relatedness [2], and, the ten type strains with the smallest MASH distances chosen per user genome. Second, an additional set of ten closely related type strains was determined via the 16S rDNA gene sequences. These were extracted from the user genomes using RNAmmer [3] and each sequence was subsequently BLASTed [4] against the 16S rDNA gene sequence of each of the currently 15554 type strains available in the TYGS database. This was used as a proxy to find the best 50 matching type strains (according to the bitscore) for each user genome and to subsequently calculate precise distances using the Genome BLAST Distance Phylogeny approach (GBDP) under the algorithm 'coverage' and distance formula d_5 [5]. These distances were finally used to determine the 10 closest type strain genomes for each of the user genomes.

Pairwise comparison of genome sequences

For the phylogenomic inference, all pairwise comparisons among the set of genomes were conducted using GBDP and accurate intergenomic distances inferred under the algorithm 'trimming' and distance formula d_5 [5]. 100 distance replicates were calculated each. Digital DDH values and confidence intervals were calculated using the recommended settings of the GGDC 2.1 [5].

Phylogenetic inference

The resulting intergenomic distances were used to infer a balanced minimum evolution tree with branch support via FASTME 2.1.6.1 including SPR postprocessing [6]. Branch support was inferred from 100 pseudo-bootstrap replicates each. The trees were rooted at the midpoint [7] and visualized with PhyD3 [8].

Type-based species and subspecies clustering

The type-based species clustering using a 70% dDDH radius around each of the 14 type strains was done as previously described [1]. The resulting groups are shown in Table 1 and 4. Subspecies clustering was done using a 79% dDDH threshold as previously introduced [9].

Results

Type-based species and subspecies clustering

The resulting species and subspecies clusters are listed in Table 4, whereas the taxonomic identification of the query strains is found in Table 1. Briefly, the clustering yielded 12 species clusters and the provided query strains were assigned to 1 of these. Moreover, user strains were located in 1 of 13 subspecies clusters.

Figure caption SSU tree

Figure 1. Tree inferred with FastME 2.1.6.1 [6] from GBDP distances calculated from 16S rDNA gene sequences. The branch lengths are scaled in terms of GBDP distance formula d_5. The numbers above branches are GBDP pseudo-bootstrap support values > 60 % from 100 replications, with an average branch support of 69.4 %. The tree was rooted at the midpoint [7].

Figure caption genome tree

Figure 2. Tree inferred with FastME 2.1.6.1 [6] from GBDP distances calculated from genome sequences. The branch lengths are scaled in terms of GBDP distance formula d_5. The numbers above branches are GBDP pseudo-bootstrap support values > 60 % from 100 replications, with an average branch support of 95.7 %. The tree was rooted at the midpoint [7].

References

[1] Meier-Kolthoff JP, Göker M. TYGS is an automated high-throughput platform for state-of-the-art genome-based taxonomy. Nat. Commun. 2019;10: 2182. DOI: 10.1038/s41467-019-10210-3

[2] Ondov BD, Treangen TJ, Melsted P, et al. Mash: Fast genome and metagenome distance estimation using MinHash. Genome Biol 2016;17: 1–14. DOI: 10.1186/s13059-016-0997-x

[3] Lagesen K, Hallin P. RNAmmer: consistent and rapid annotation of ribosomal RNA genes. Nucleic Acids Res. Oxford Univ Press; 2007;35: 3100–3108. DOI: 10.1093/nar/gkm160

[4] Camacho C, Coulouris G, Avagyan V, Ma N, Papadopoulos J, Bealer K, et al. BLAST+: architecture and applications. BMC Bioinformatics. 2009;10: 421. DOI: 10.1186/1471-2105-10-421

[5] Meier-Kolthoff JP, Auch AF, Klenk H-P, Göker M. Genome sequence-based species delimitation with confidence intervals and improved distance functions. BMC Bioinformatics. 2013;14: 60. DOI: 10.1186/1471-2105-14-60

[6] Lefort V, Desper R, Gascuel O. FastME 2.0: A comprehensive, accurate, and fast distance-based phylogeny inference program. Mol Biol Evol. 2015;32: 2798–2800. DOI: 10.1093/molbev/msv150

[7] Farris JS. Estimating phylogenetic trees from distance matrices. Am Nat. 1972;106: 645–667.

[8] Kreft L, Botzki A, Coppens F, Vandepoele K, Van Bel M. PhyD3: A phylogenetic tree viewer with extended phyloXML support for functional genomics data visualization. Bioinformatics. 2017;33: 2946–2947. DOI: 10.1093/bioinformatics/btx324

[9] Meier-Kolthoff JP, Hahnke RL, Petersen J, Scheuner C, Michael V, Fiebig A, et al. Complete genome sequence of DSM 30083^{T}, the type strain (U5/41^{T}) of *Escherichia coli*, and a proposal for delineating subspecies in microbial taxonomy. Stand Genomic Sci. 2014;9: 2. DOI: 10.1186/1944-3277-9-2
