# Supplementary material for: Complete Genome Analysis of Undecylprodigiosin Pigment Biosynthesizing Marine Streptomyces Species Displaying Potential Bioactive Applications
Source: Microorganisms. 2021 Oct 28;9(11):2249. doi: 10.3390/microorganisms9112249 (PMC8618203; doi:10.3390/microorganisms9112249)

Sup. Data5: All clusters and their similarity to the other Streptomyces.(Please click the link below to access the details.)
https://antismash.secondarymetabolites.org/upload/bacteria-5632323e-f894-4250-98c1-2e29200883a5/index.html#

Cluster 1:

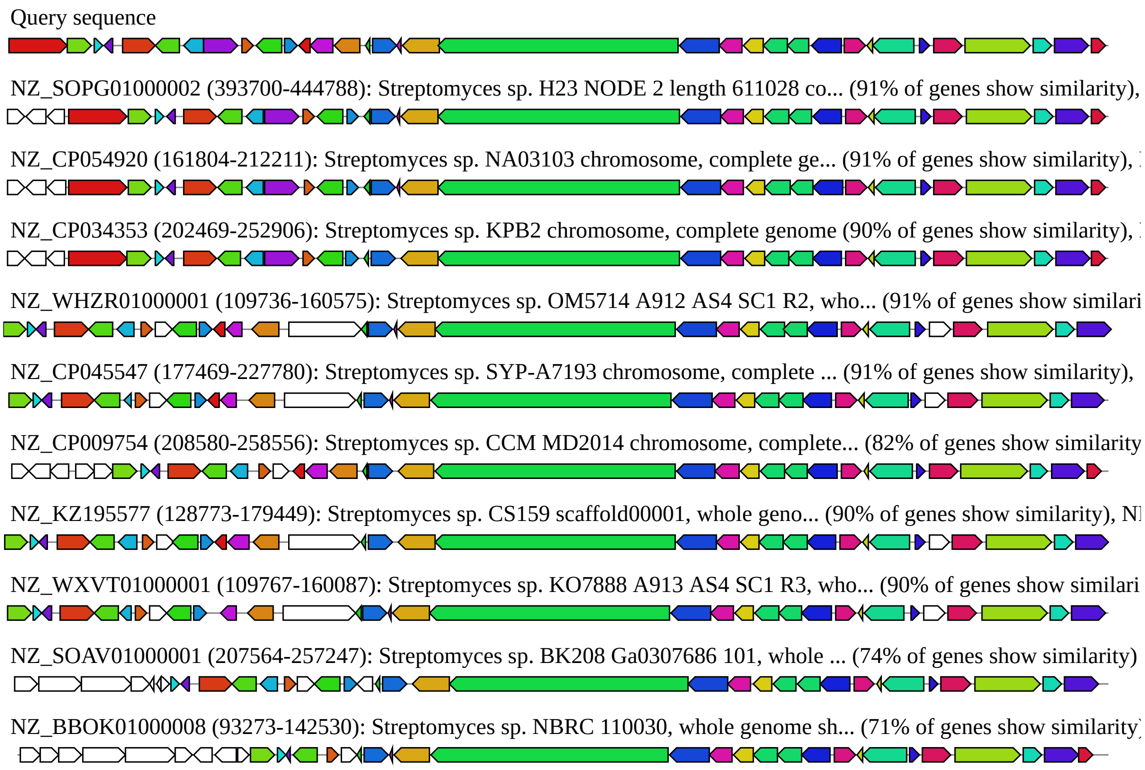

Cluster 2:

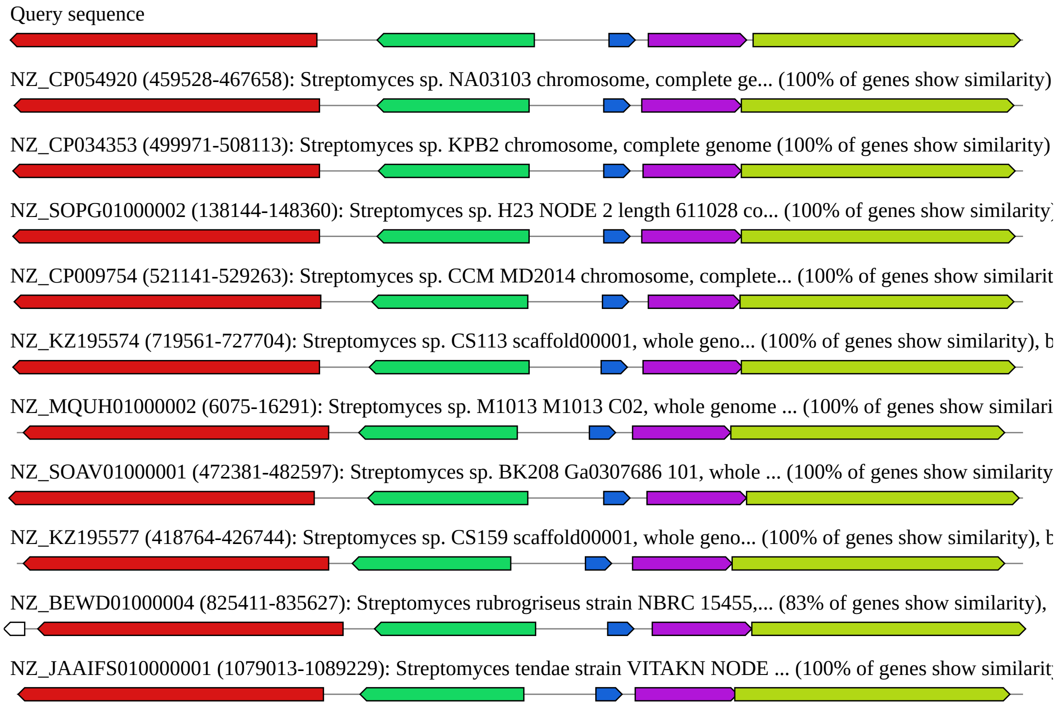

Cluster 3:

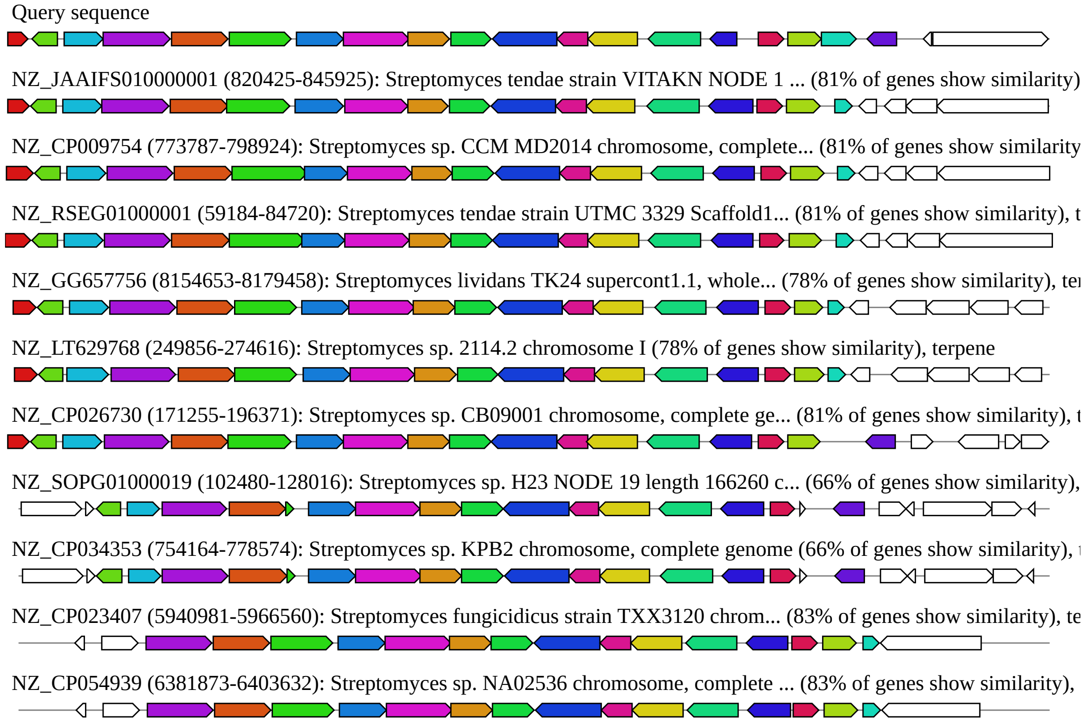

Cluster 4:

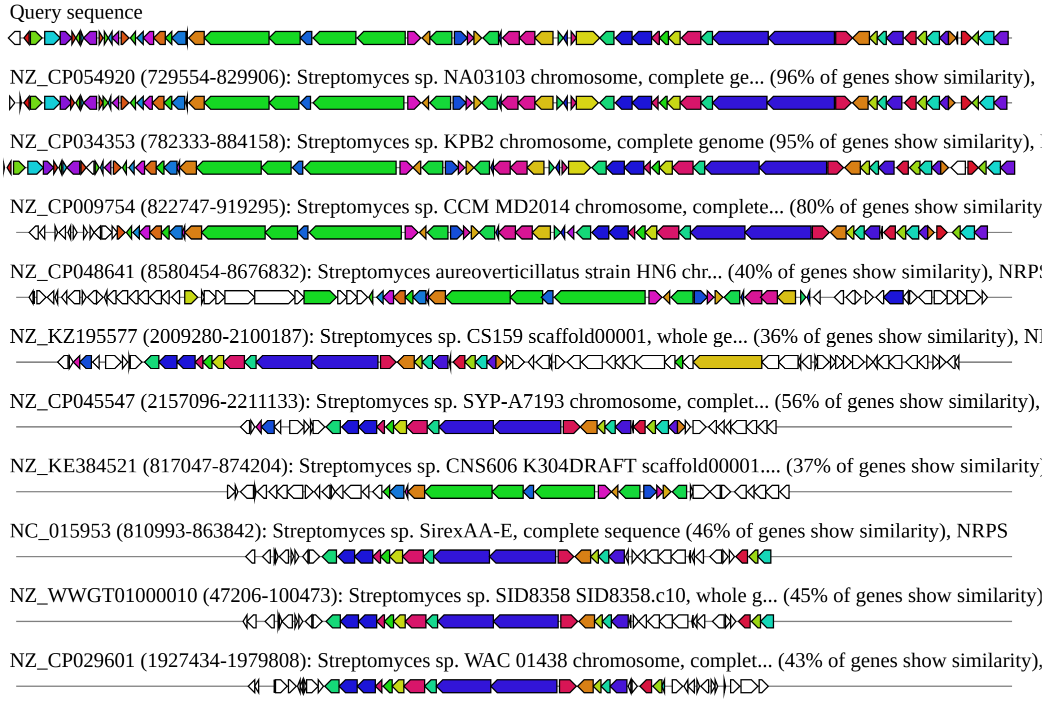

Cluster 5:

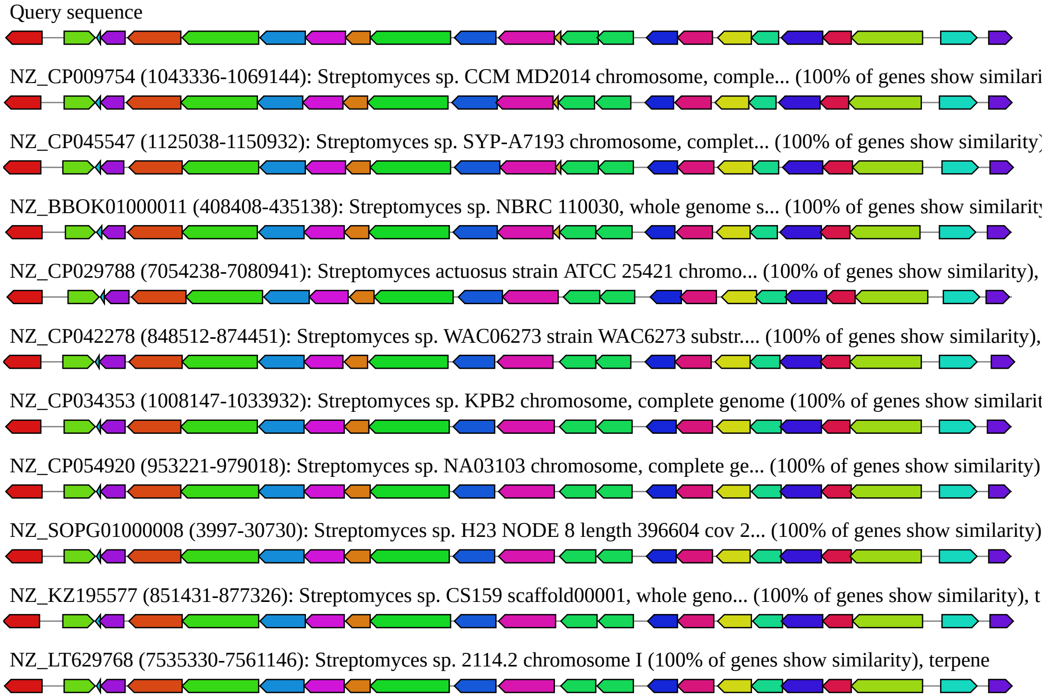

Cluster 6:

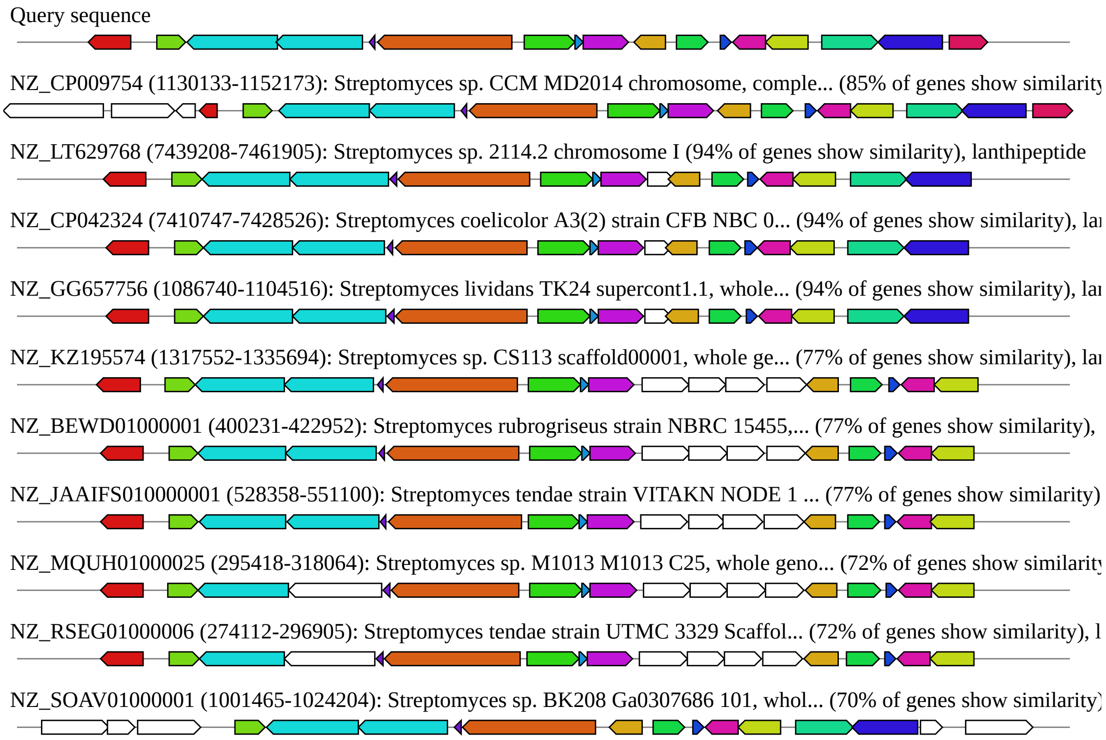

Cluster 7:

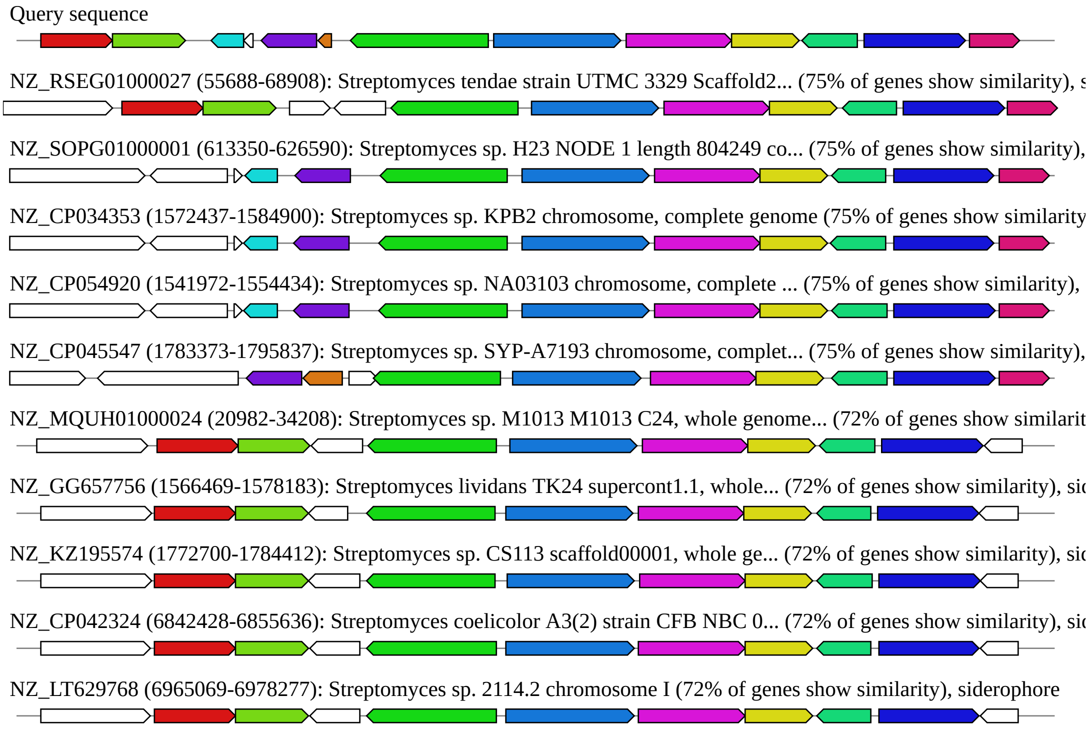

Cluster 8:

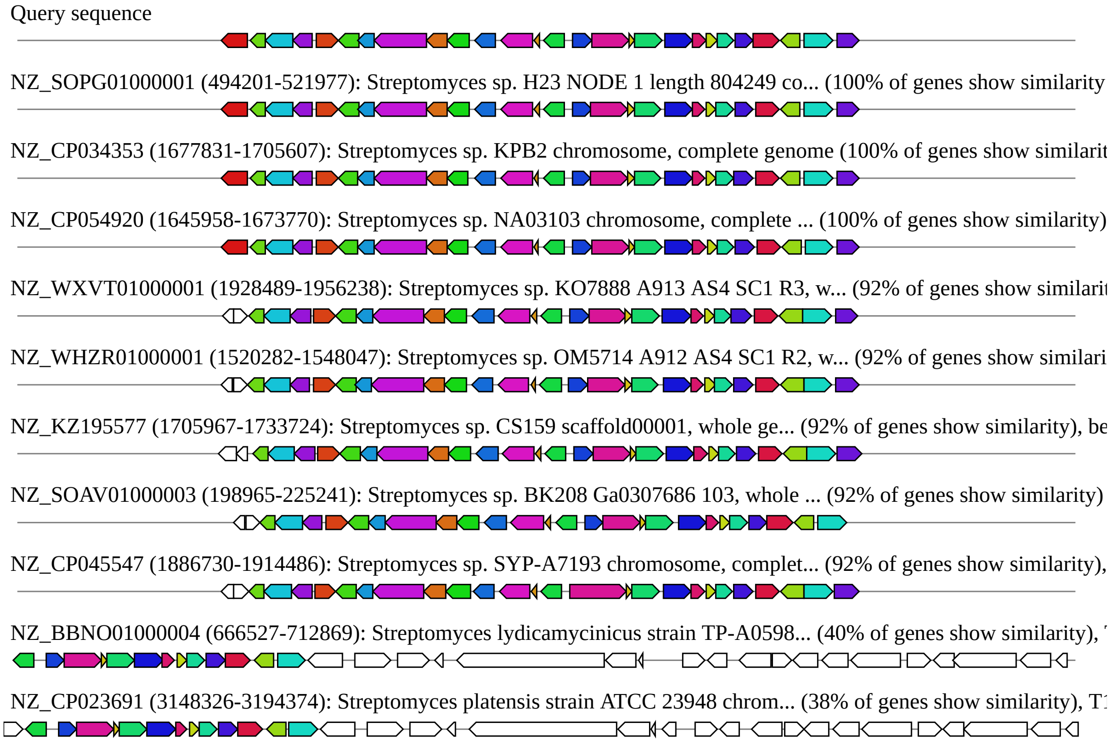

Cluster 9:

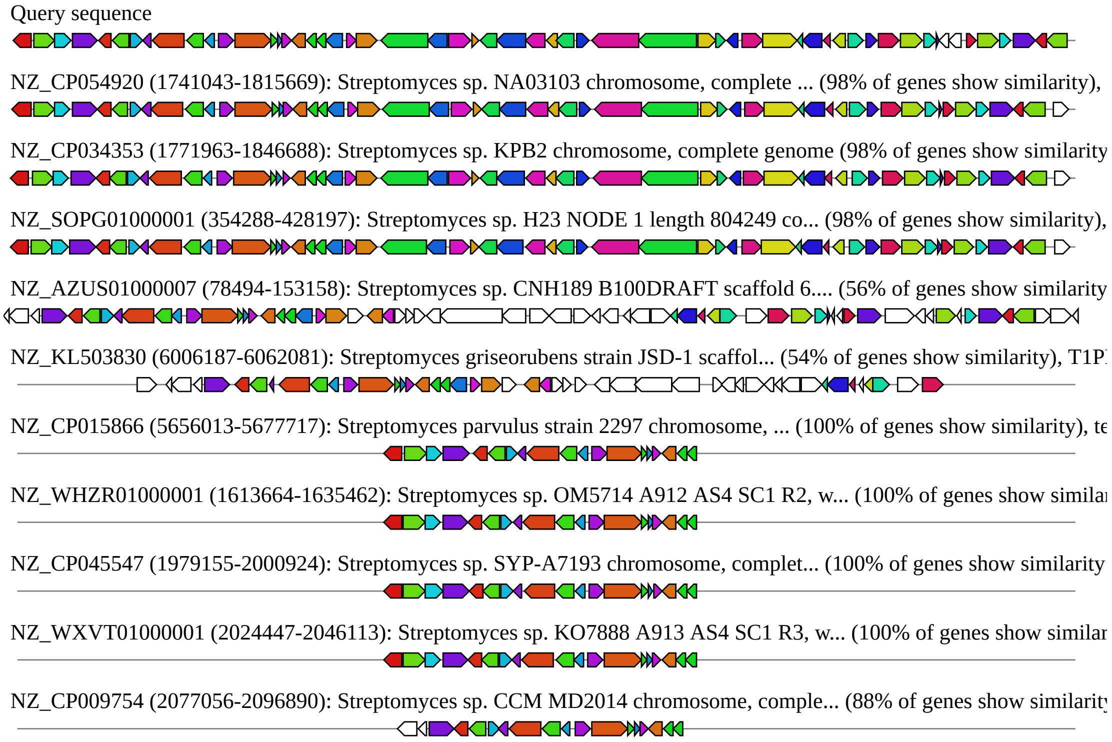

Cluster 10:

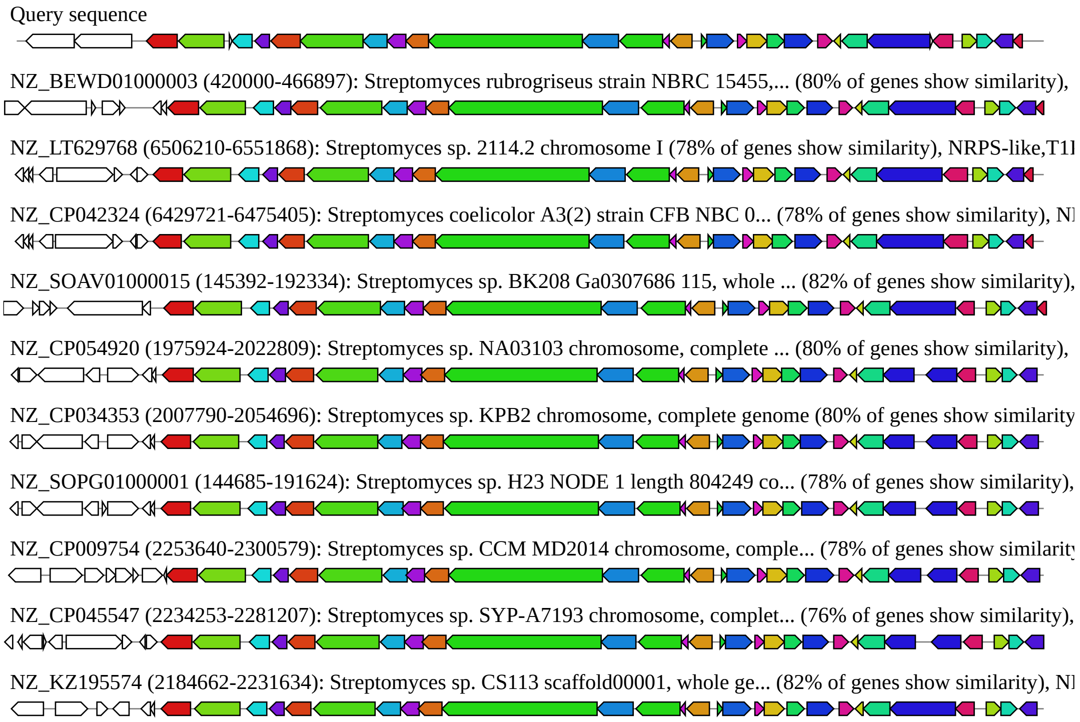

Cluster 11:

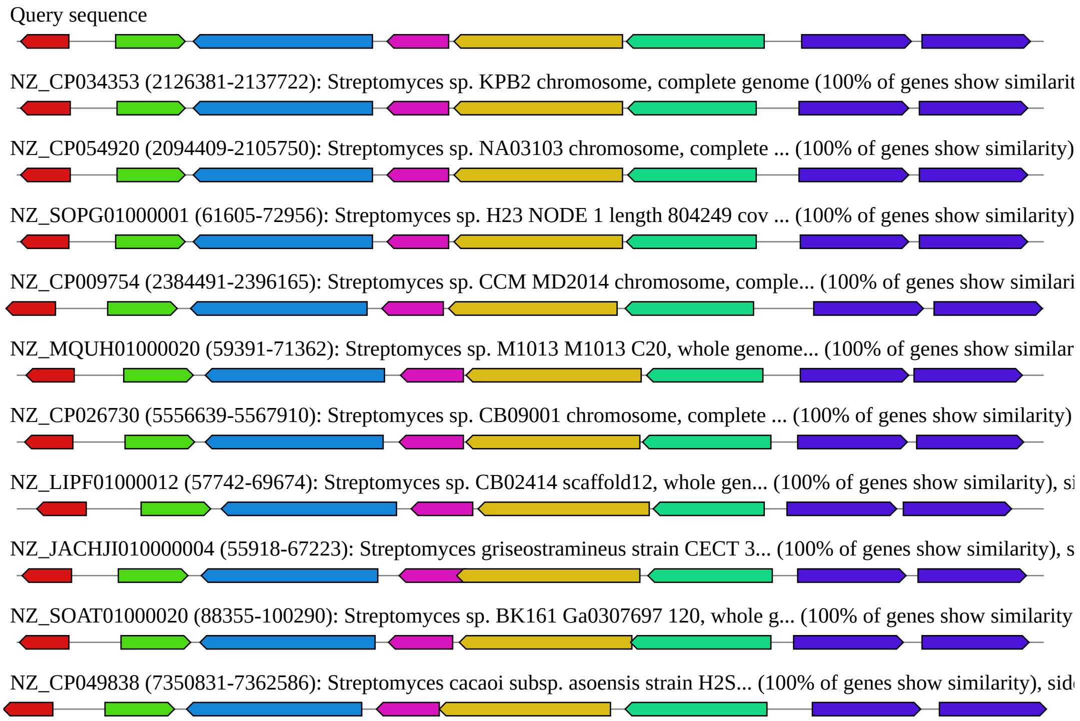

Cluster 12:

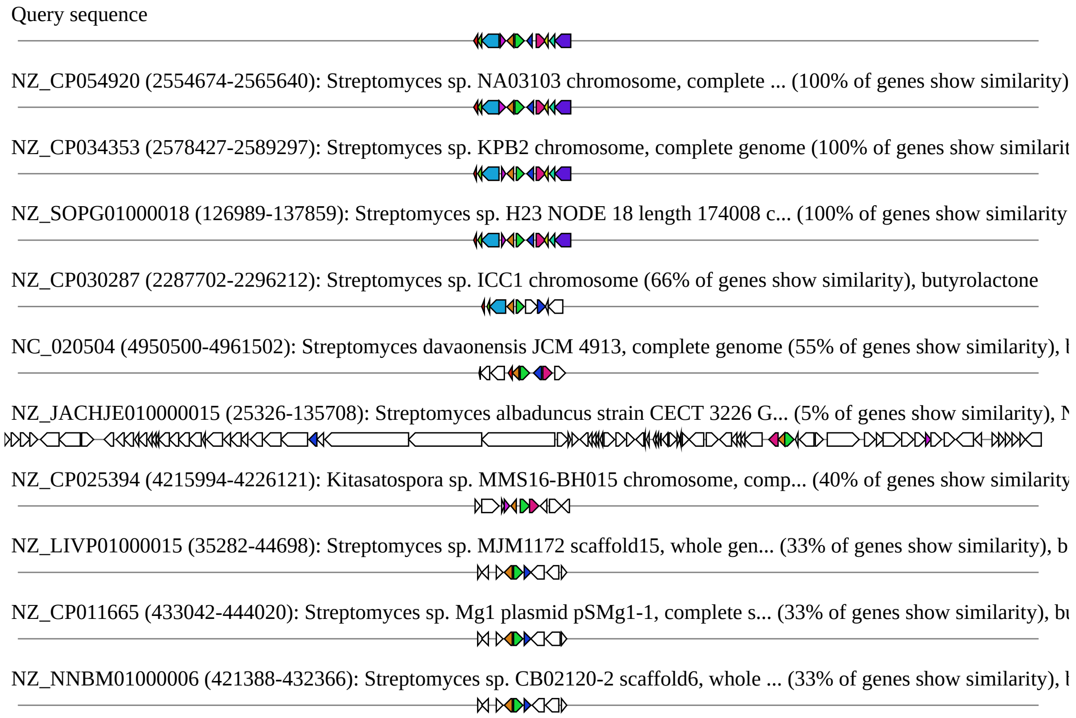

Cluster 13:

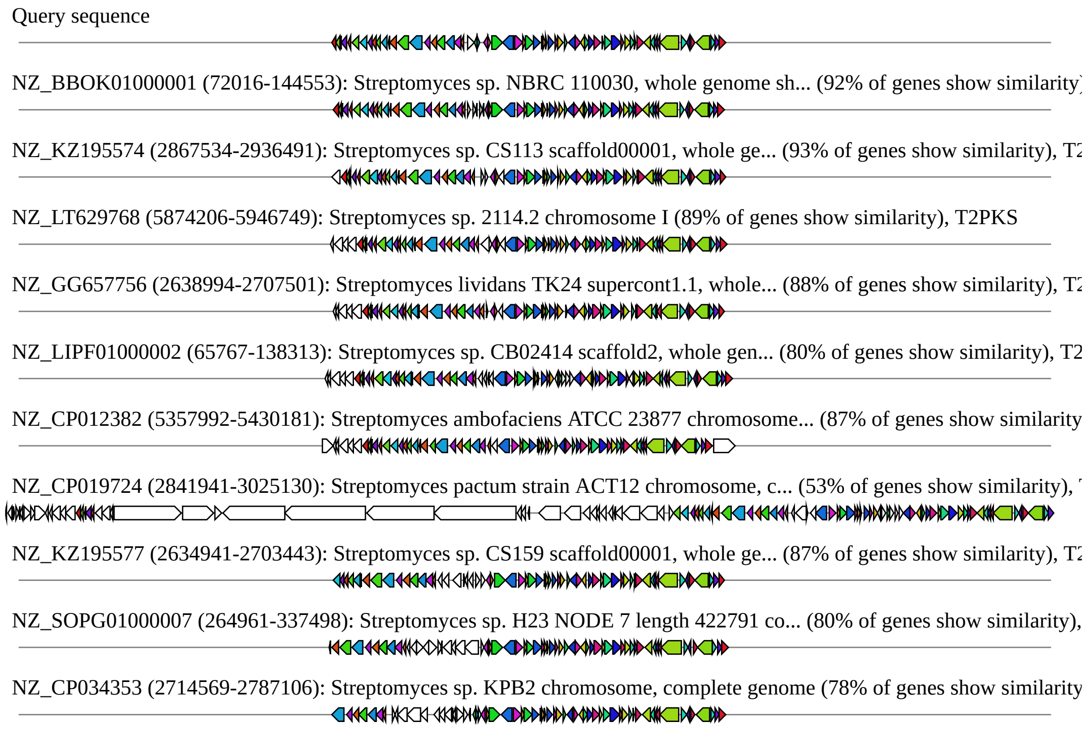

Cluster 14:

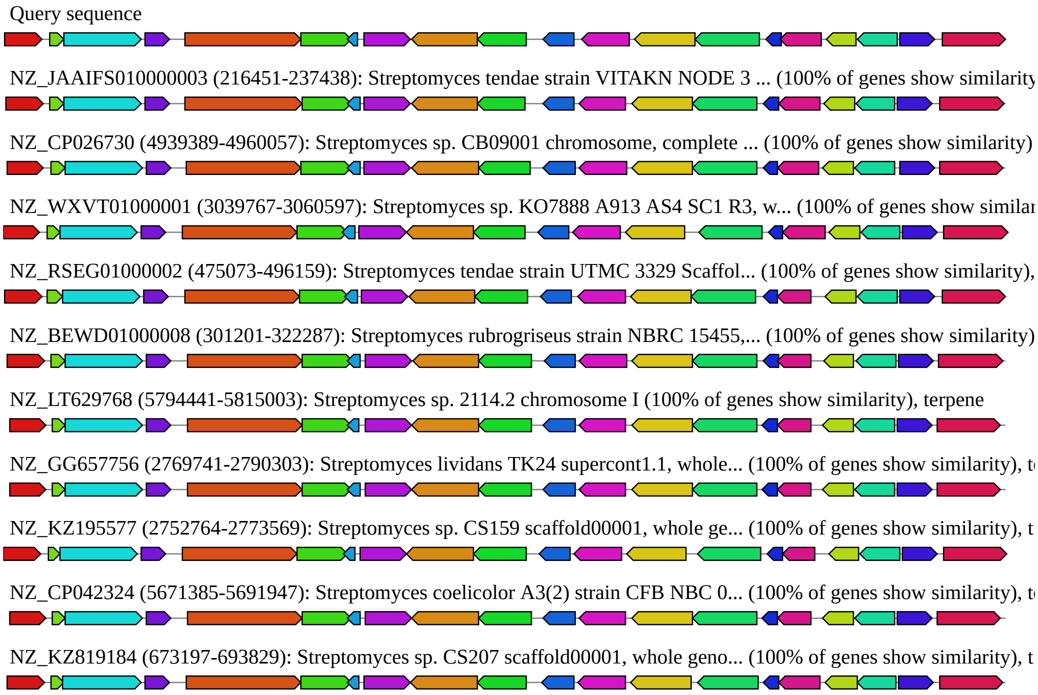

Cluster 15:

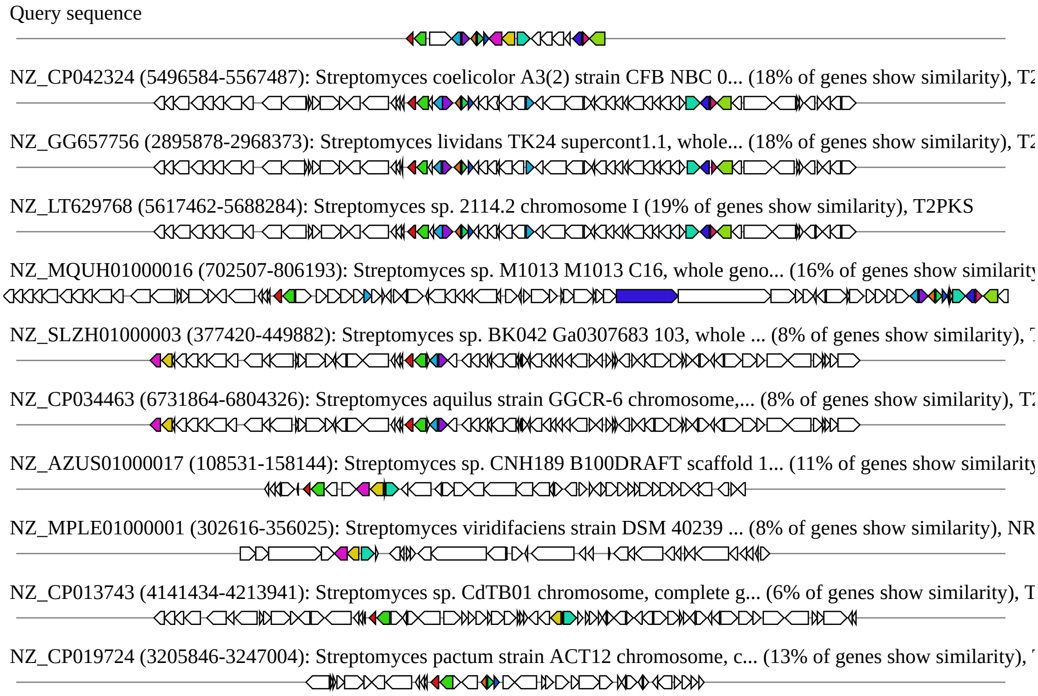

Cluster 16:

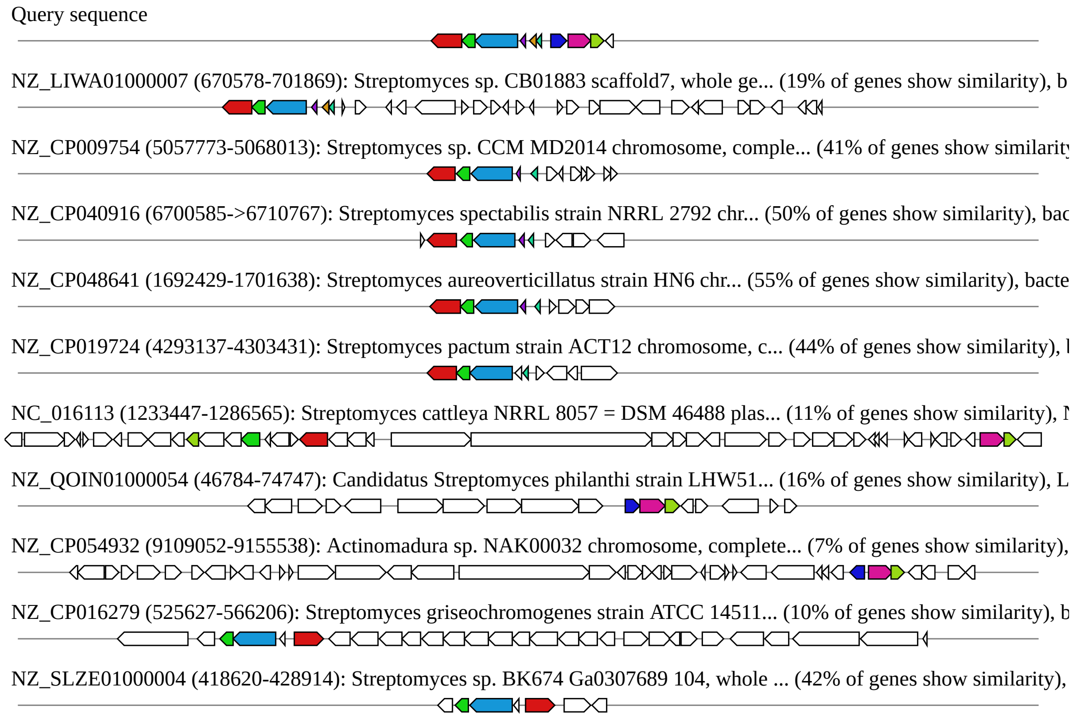

Cluster 17:

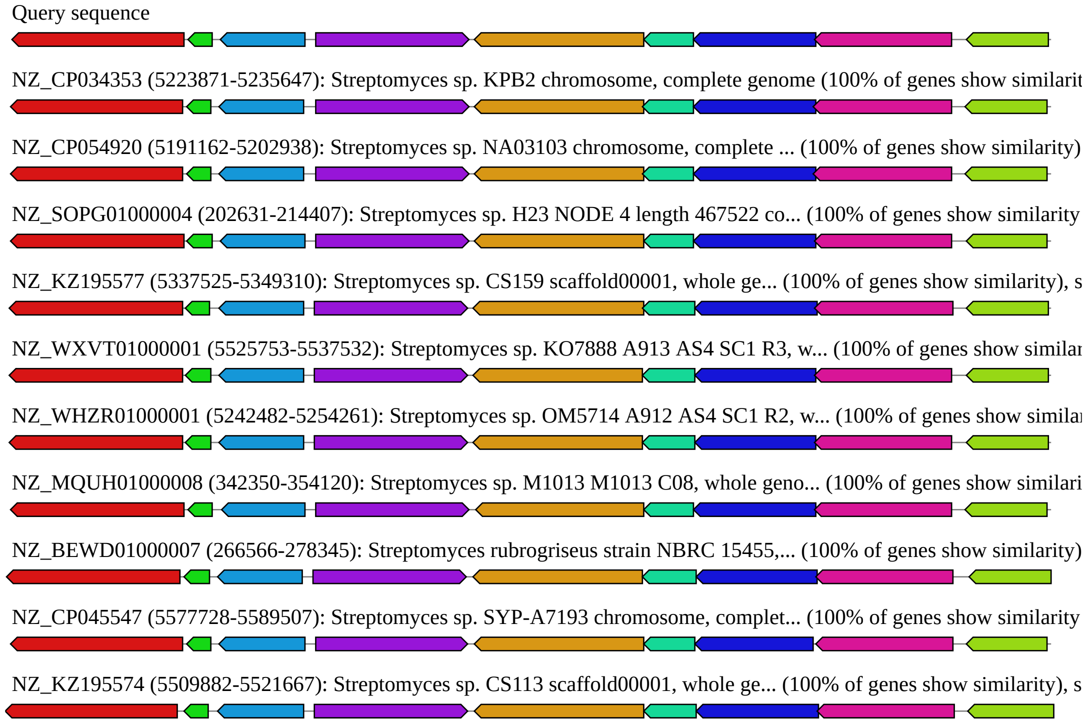

Cluster 18:

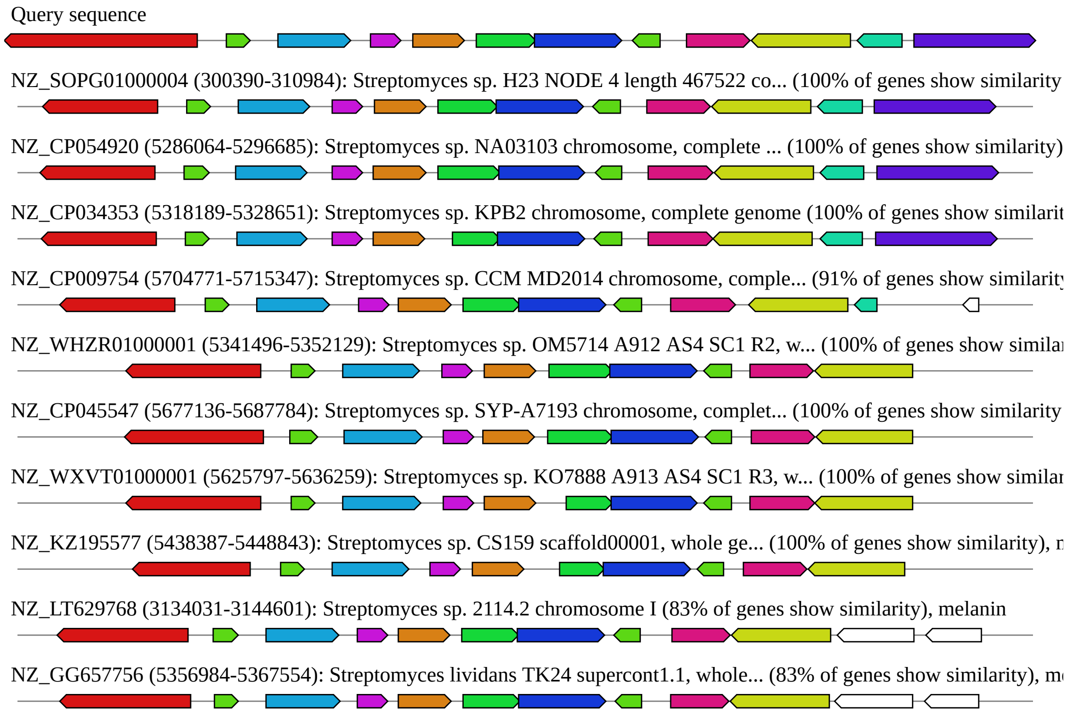

Cluster 19:

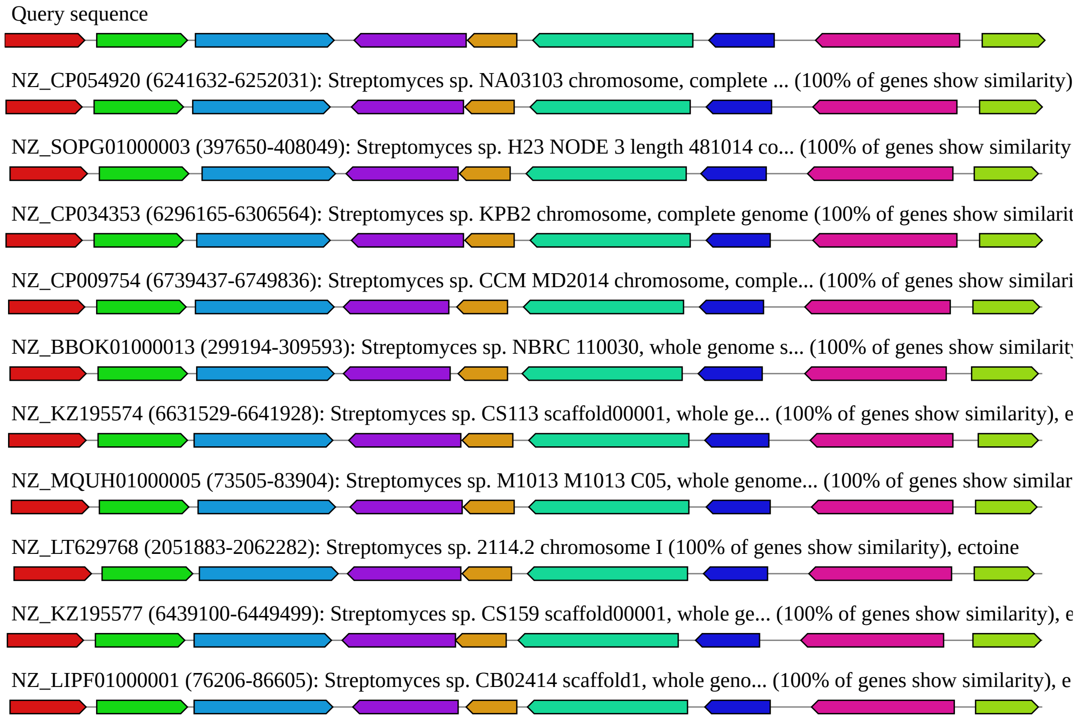

Cluster 20:

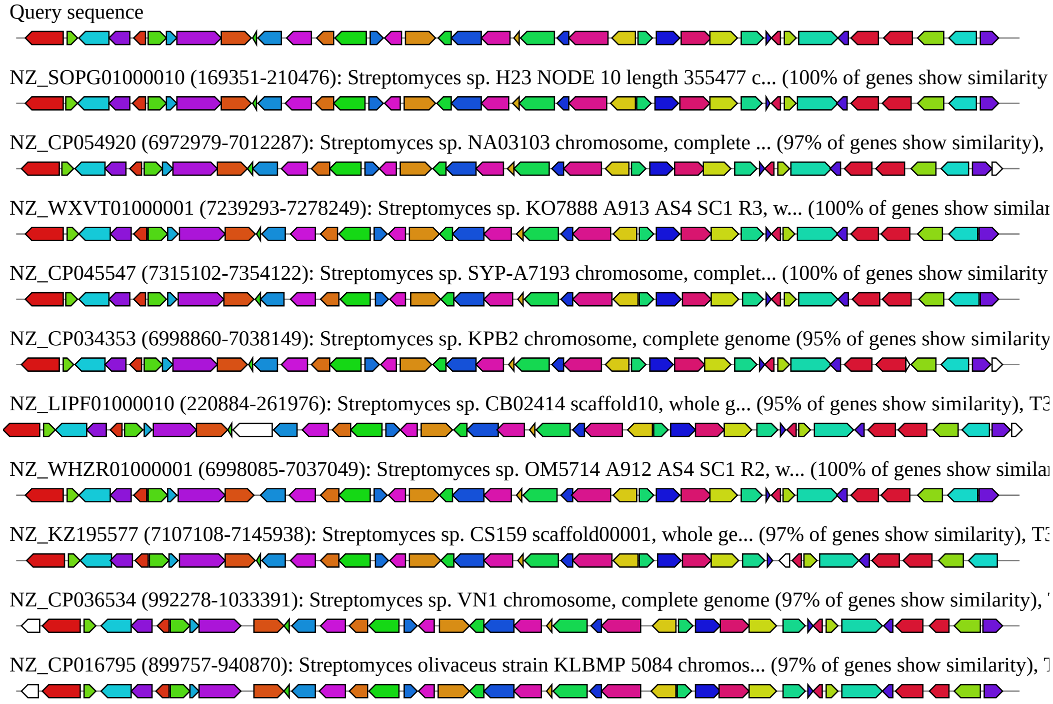

Cluster 21:

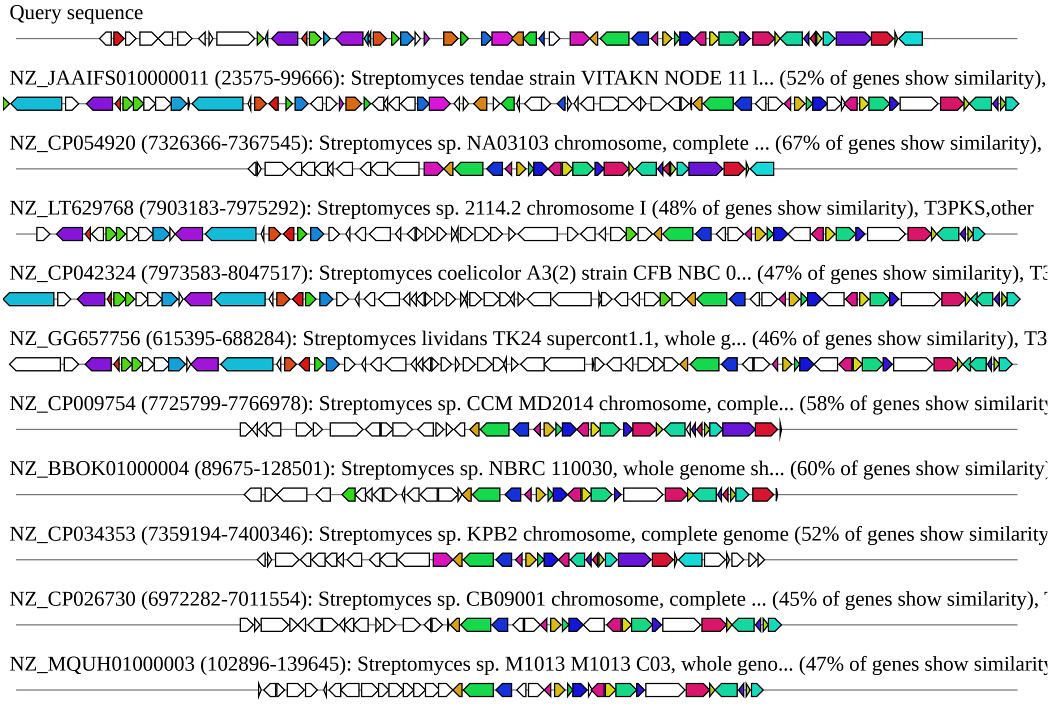

Cluster 22:

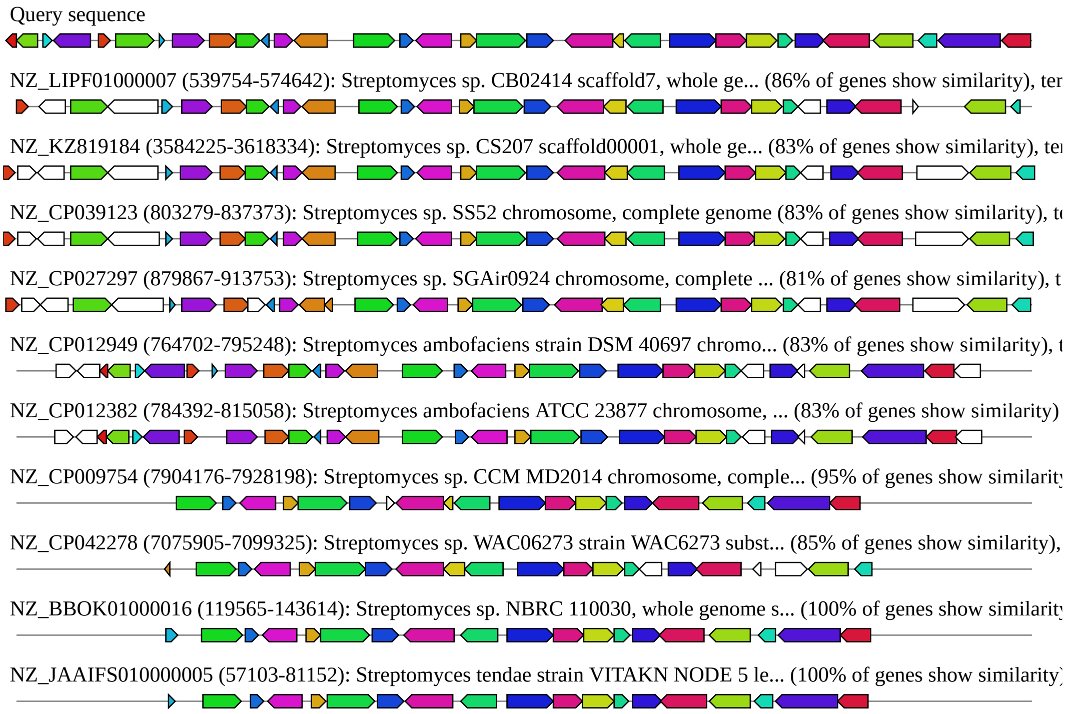

Cluster 23:

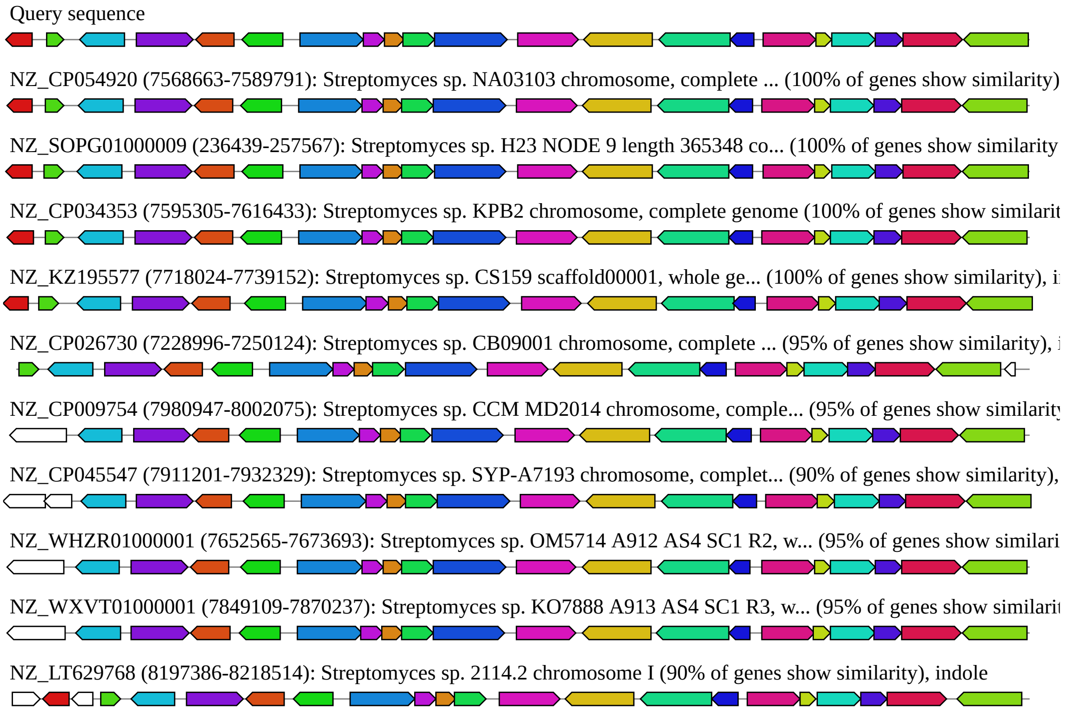

Supplement: Supplementary file 1 [file microorganisms-09-02249-s001.zip › Sup Data5.pdf]
