# Supplementary material for: Complete Genome Analysis of Undecylprodigiosin Pigment Biosynthesizing Marine Streptomyces Species Displaying Potential Bioactive Applications
Source: Microorganisms. 2021 Oct 28;9(11):2249. doi: 10.3390/microorganisms9112249 (PMC8618203; doi:10.3390/microorganisms9112249)
Supplement: Supplementary file 1 [file microorganisms-09-02249-s001.zip › Supplementary figures.pdf]

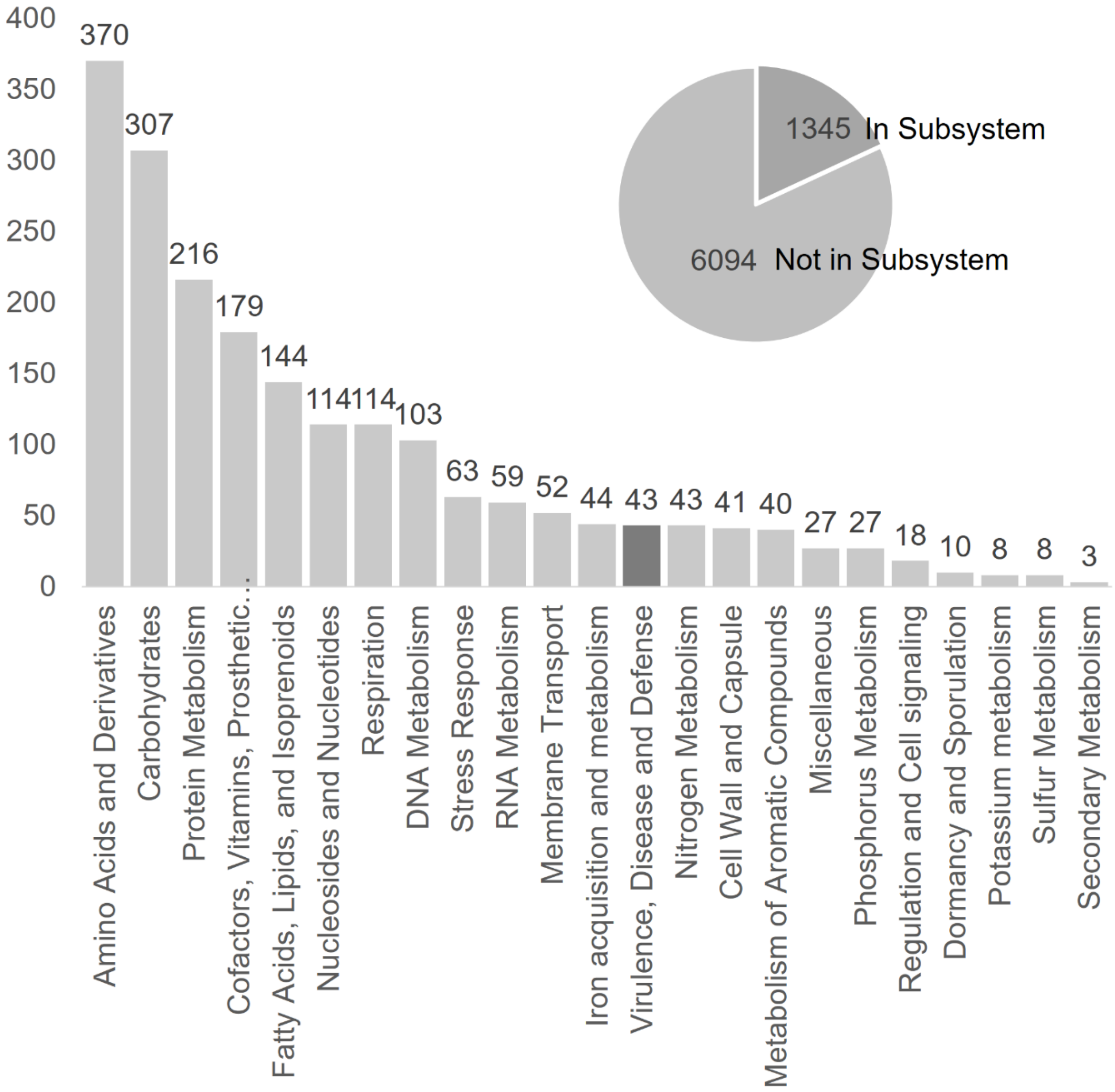

Figure S1: Subsystem coverage of the strain BSE6.1 genes

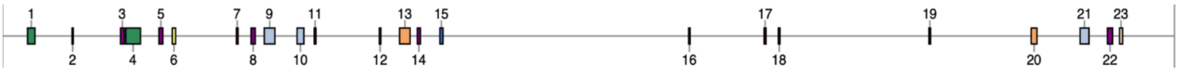

Region	Type	From	To	Most similar known cluster		Similarity
Region 1	NRPS	171,104	221,425	coelichelin	NRP	100%
Region 2	RiPP-like	475,707	483,910	informatipeptin	RiPP:Lanthipeptide	42%
Region 3	terpene	807,542	832,634	isorenieratene	Terpene	100%
Region 4	NRPS	843,405	944,099	coelibactin	NRP	100%
Region 5	terpene	1,071,114	1,096,932	hopene	Terpene	100%
Region 6	lanthipeptide-class-iii	1,162,010	1,184,778	SapB	RiPP:Lanthipeptide	100%
Region 7	siderophore	1,600,623	1,612,356	enduracidin	NRP	4%
Region 8	betalactone	1,702,188	1,729,972	hormaomycin /A1-A6	NRP:Cyclic depsipeptide	13%
Region 9	terpene,NRPS,NRPS-like,RiPP-like	1,791,691	1,864,931	ashimides	NRP	100%
Region 10	T1PKS,NRPS-like,prodigiosin	2,016,202	2,063,074	undecylprodigiosin	NRP + Polyketide	100%
Region 11	siderophore	2,134,318	2,145,693			
Region 12	butyrolactone	2,579,402	2,590,367			
Region 13	T2PKS	2,718,175	2,790,711	spore pigment	Polyketide	66%
Region 14	terpene	2,840,156	2,860,898	albaflavenone	Terpene	100%
Region 15	RRE-containing	2,994,252	3,015,571	thiotetroamide	Polyketide	11%
Region 16	RiPP-like	4,697,728	4,708,021			
Region 17	siderophore	5,215,285	5,227,060	desferrioxamin B / desferrioxamine E	Other	83%
Region 18	melanin	5,312,530	5,323,123	melanin	Other	60%
Region 19	ectoine	6,343,949	6,354,347	ectoine	Other	100%
Region 20	T3PKS	7,043,953	7,082,950	herboxidiene	Polyketide	8%
Region 21	other,T3PKS	7,378,897	7,438,864	germicidin	Other	100%
Region 22	terpene	7,565,664	7,600,619	carotenoid	Terpene	45%
Region 23	indole	7,648,350	7,669,477	5-isoprenylindole-3-carboxylate β-D-glycosyl ester	Other	28%

Figure S2: Gene clusters of secondary metabolites in the BSE6.1 genome

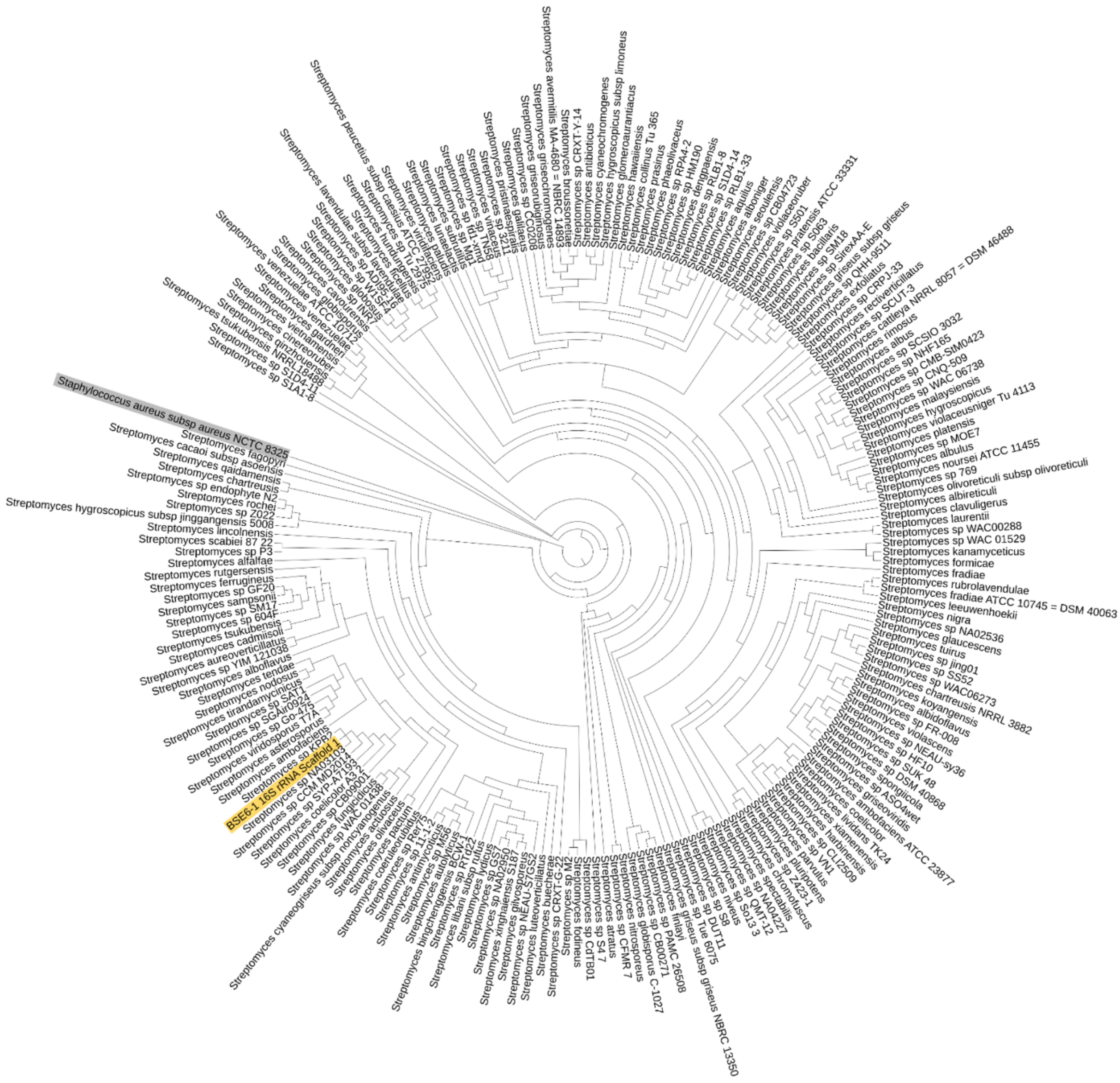

Figure S3: Maximum-Likelihood tree of16S rRNA genes belonging to 208 Streptomyces species and strain BSE6.1. Outgroup: Staphylococcus aureus, Bootstrap value: 1000.

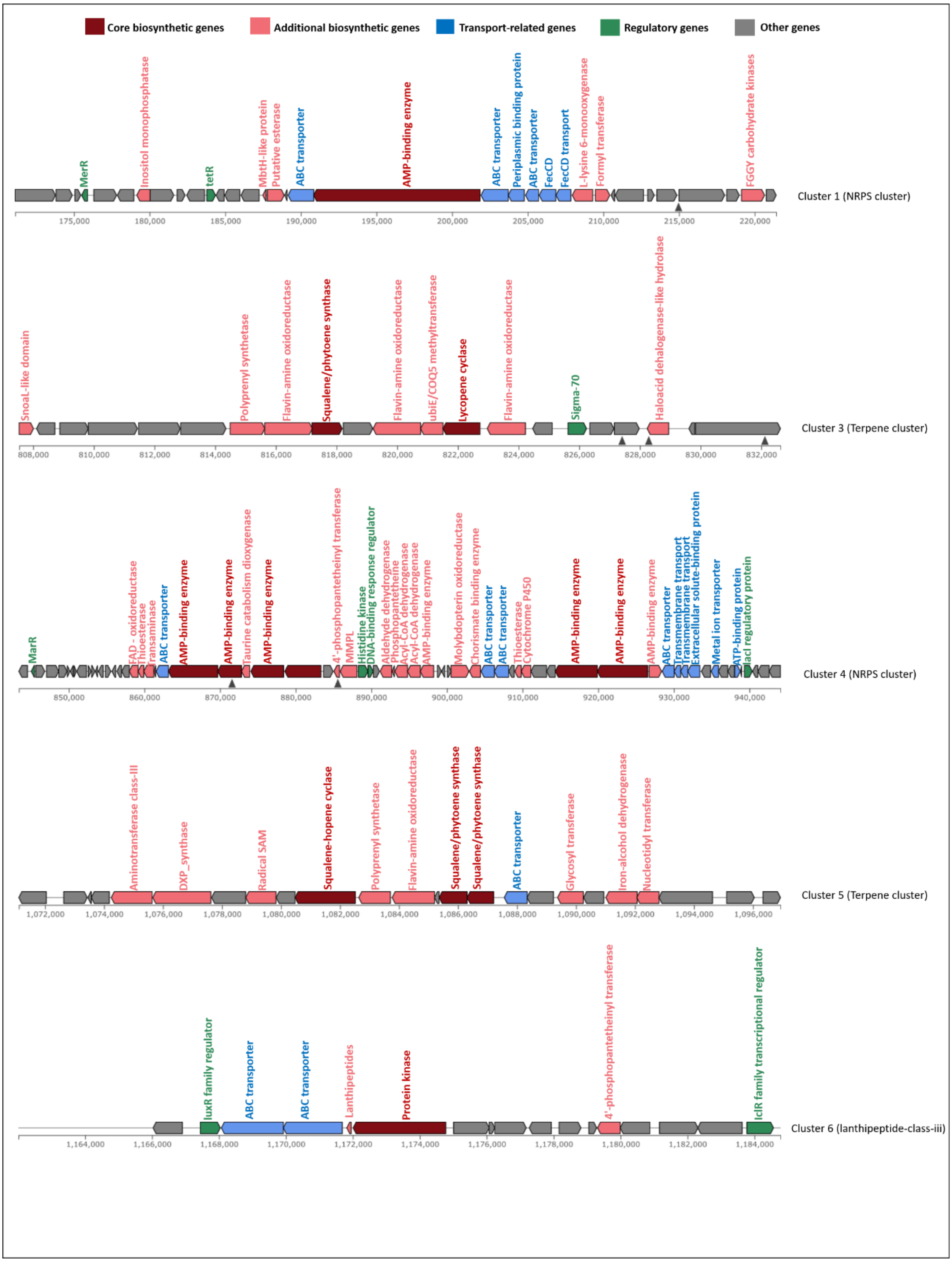

Figure S4: Details of gene clusters which are >75% similar to existing ones.

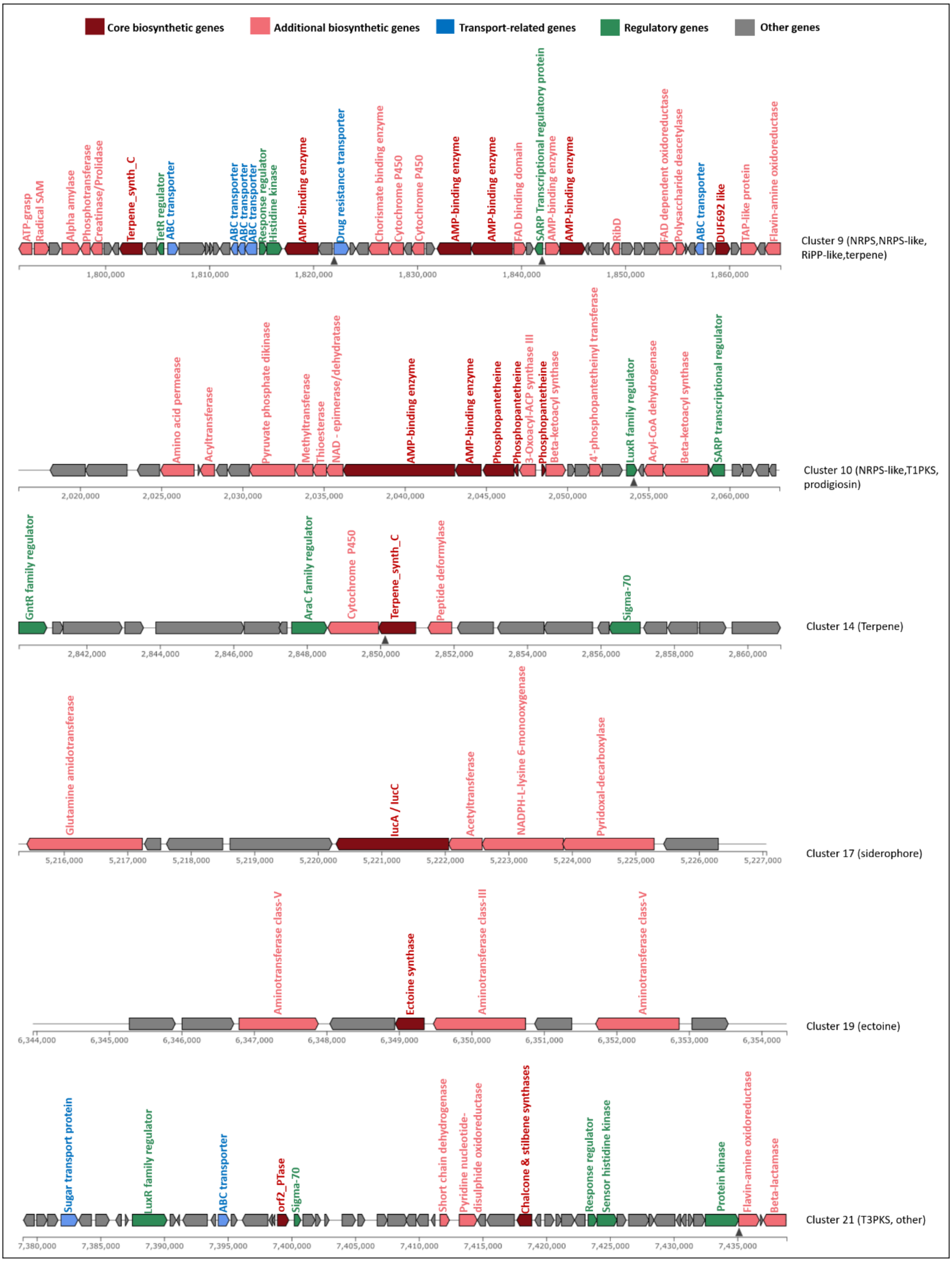

Figure S5. Details of gene clusters which are >75% similar to existing ones.
